# The effectiveness of interventions to reduce adverse outcomes among older adults following Emergency Department discharge: umbrella review

**DOI:** 10.1186/s12877-022-03007-5

**Published:** 2022-05-28

**Authors:** Mairéad Conneely, Siobhán Leahy, Liz Dore, Dominic Trépel, Katie Robinson, Fionnuala Jordan, Rose Galvin

**Affiliations:** 1 School of Allied Health, Faculty of Education and Health Sciences, Ageing Research Centre, Health Research Institute, University of Limerick, Limerick, Ireland; 2 Glucksman Library, Education & Health Sciences, University of Limerick, Limerick, Ireland; 3 Present Address: Department of Sport, Exercise & Nutrition, School of Science & Computing, Atlantic Technological University, ATU Galway City, Galway, Ireland; 4 Trinity Institute of Neurosciences, School of Medicine, Trinity College Dublin, Dublin, Ireland; 5 School of Nursing and Midwifery, College of Medicine, Nursing and Health Sciences, National University of Ireland Galway, University Road, Galway, Ireland

**Keywords:** Older adults, Emergency department, Geriatrics, Evidence synthesis, Umbrella review

## Abstract

**Background:**

Population ageing is increasing rapidly worldwide. Older adults are frequent users of health care services including the Emergency Department (ED) and experience a number of adverse outcomes following an ED visit. Adverse outcomes include functional decline, unplanned hospital admission and an ED revisit. Given these adverse outcomes a number of interventions have been examined to improve the outcomes of older adults following presentation to the ED. The aim of this umbrella review was to evaluate the effectiveness of ED interventions in reducing adverse outcomes in older adults discharged from the ED.

**Methods:**

Systematic reviews of randomised controlled trials investigating ED interventions for older adults presenting to the ED exploring clinical, patient experience and healthcare utilisation outcomes were included. A comprehensive search strategy was employed in eleven databases and the PROSPERO register up until June 2020. Grey literature was also searched. Quality was assessed using the A MeaSurement Tool to Assess Systematic Reviews 2 tool. Overlap between systematic reviews was assessed using a matrix of evidence table. An algorithm to assign the Grading of Recommendations Assessment, Development and Evaluation to assess the strength of evidence was applied for all outcomes.

**Results:**

Nine systematic reviews including 29 randomised controlled trials were included. Interventions comprised of solely ED-based or transitional interventions. The specific interventions delivered were highly variable. There was high overlap and low methodological quality of the trials informing the systematic reviews. There is low quality evidence to support ED interventions in reducing functional decline, improving patient experience and improving quality of life. The quality of evidence of the effectiveness of ED interventions to reduce mortality and ED revisits varied from very low to moderate. Results were presented narratively and summary of evidence tables created.

**Conclusion:**

Older adults are the most important emerging group in healthcare for several economic, social and political reasons. The existing evidence for the effectiveness of ED interventions for older adults is limited. This umbrella review highlights the challenge of synthesising evidence due to significant heterogeneity in methods, intervention content and reporting of outcomes. Higher quality intervention studies in line with current geriatric medicine research guidelines are recommended, rather than the publication of further systematic reviews.

**Trial registration:**

UMBRELLA REVIEW REGISTRATION: PROSPERO (CRD42020145315).

**Supplementary Information:**

The online version contains supplementary material available at 10.1186/s12877-022-03007-5.

## Introduction

Global demographics indicate that most populations are ageing around the world [1–5]. This demographic shift presents both opportunities and challenges. Ageing is often associated with multimorbidity [6–8] and reduced functional capacity [9]. As a result, older adults are frequent users of health care services [10–13], attributing for up to one quarter of all Emergency Department (ED) attendees [14–16]. Changes in family demographics, a lack of aged-care facilities, functional and cognitive impairments, social problems and problems with accessing primary care services have been proposed as explaining why more older adults are seeking ED services [7, 17–20].

EDs are complex and challenging environments to provide care to older adults [21, 22]. Older adults present with complex health complaints [23], consume significant ED staff time [24] with heterogeneous clinical and social care needs compared to other ED patients [16, 23, 25, 26]. Between 45 to 60% of older adults presenting to the ED will be discharged directly to their own home [27, 28]. Evidence demonstrates that older adults experience high rates of adverse outcomes post discharge from the ED [29–31] as they experience a period of increased vulnerability following presentation to, and consequent discharge from, the ED [23, 32, 33]. A systematic review of 32 prospective and retrospective cohort studies reported that approximately 20% of older adults discharged from the ED return within 30 days [34], while 10–45% of older adults experience functional decline at 3 months post ED visit [35]. Furthermore, there is a high rate of institutionalisation following ED discharge and older adults have a higher rate of mortality than younger age groups post ED discharge. Older adults who return to the ED within 7 days and within 30 days following initial ED index visit, often return with the same presenting complaint again [15], indicating that a lack of continuity of care may be a reason for this increase in acute health care utilisation [30].

In light of the high rate of returns to the ED, in addition to other adverse outcomes following an index visit, a range of interventions have been examined to improve the outcomes of older adults following presentation to the ED [2, 36, 37]. These include increased ED staffing, implementation of care pathways based on risk assessment, screening tools, geriatric nurse led interventions, comprehensive geriatric assessment, integrated care case management, within the ED and post-discharge, and discharge planning [2, 38, 39]. For example, Karam et al. (2015) [40] reported that ED-based interventions that extended beyond the ED and those with an integrated model of care may lead to improved outcomes including reduced nursing home admission, ED revisits, hospitalisation and death. Hughes et al. [36] investigated the impact of interventions that were delivered during the ED visit, post ED discharge and integrated care that bridged the ED and the home using a variety of strategies (case management, management/medication safety and discharge planning). The authors reported that the interventions were heterogeneous with a mixed pattern of effectiveness on clinical and service outcomes. In contrast to the Karam et al. (2015) review [40], the authors suggested a small positive effect of ED interventions on functional status but no effect on other outcomes including patient experience, quality of life, rates of hospitalisation at or after the initial ED index visit, or rates of return to the ED. Indeed a scoping review to identify evidence for the identification and management of frail older adults in the ED published in 2017 recommended a thorough synthesis of the evidence to inform practice [41].

A preliminary scoping search revealed that numerous systematic reviews evaluated the effectiveness of ED interventions on reducing adverse outcomes in older adults. Given the number of systematic reviews published in this area, an umbrella review is warranted with aim of evaluating the effectiveness of ED interventions in reducing adverse outcomes in older adults discharged from the ED. An umbrella review is conducted to summarise the evidence from multiple systematic reviews to address a broad evidence base and research question [42–46]. The unit of analysis in an umbrella review is a systematic review thus providing an opportunity to compare and contrast the findings of multiple systematic reviews [44, 47]. A recent umbrella review [48] summarised which ED interventions met the needs of older adults and reported no individual intervention was more beneficial. This umbrella review included systematic reviews and non-systematic reviews of randomised controlled trials and observational studies which focused on the intervention delivered within the ED as well as in the inpatient setting. The overall aim of our umbrella review was to explore the effectiveness of interventions based in the ED or initiated in the ED, and transitional interventions for older adults discharged home from the ED, as well as the outcomes reported in systematic reviews.

The objectives of this umbrella review were to:

1. Identify, appraise and synthesise all relevant systematic reviews of ED based interventions, transitional interventions from the ED to the community and ED initiated interventions to reduce adverse outcomes in older adults following ED discharge.
2. Identify commonalities and differences between interventions with attention focusing on the characteristics of interventions, the quality of the evidence and other pertinent factors such as heterogeneity (clinical and methodological) within and across reviews.

## Materials and methods

An umbrella review was conducted to identify and synthesise the results of systematic reviews [49–51] of ED interventions for older adults discharged from the ED within 72 h of index visit. There is an absence of specific guidelines on the conduct and reporting of umbrella reviews with the Preferred Reporting Items for Overviews of Reviews (PRIOR) guidelines currently under development [52]. Therefore, in lieu of specific guidance for umbrella reviews, this umbrella review was conducted according to the Joanna Briggs Institute methodology of conducting an umbrella review [42] and key aspects of the methods and results of umbrella reviews outlined in the protocol for the PRIOR guidelines [52]. This umbrella review followed an a priori published protocol [53] registered with the International Prospective Register of Systematic Reviews (PROSPERO) on 28^th^ April 2020 (CRD42020145315).

### Inclusion criteria

Eligibility criteria for this umbrella review were established using the Population, Intervention, Comparator, Outcome and Study design (PICOS) framework:

### Population

Older adults (aged 65 years and over) presenting to the ED or Acute Medical Unit (AMU) and discharged within 72 h of index visit.

### Interventions

Interventions including ED based interventions, transitional interventions and ED initiated interventions.

### Comparator

All comparators were considered.

### Outcomes

Our primary outcome of interest was functional status or functional decline measured using a validated tool of functional ability (e.g. Activities of Daily Living, Instrumental Activities of Daily Living). Secondary outcomes focused on patient and process outcomes including Health related Quality of life (e.g. EuroQol, EQ-5D), mortality, patient experience or satisfaction (studies reporting any validated measure of patient experience and satisfaction); healthcare utilisation (three indicators of healthcare utilisation: ED revisit or readmission, hospital admission rates (following ED discharge), and ED length of stay [54].

### Study design and context

We considered quantitative systematic reviews that included randomised controlled trials (RCTs) conducted in the ED in any geographical location with or without meta-analysis and research synthesis. Systematic reviews of mixed research design were also considered for inclusion if it was possible to extract the data from RCTs. The definition of a systematic review was as follows [55, 56]:

- A research question articulated using the participants, interventions, comparisons, outcomes and study design (PICOS) format
- Criteria inclusive of all study designs
- A full search string strategy for a minimum of one electronic database (reported in supplementary material)
- A database search reported in the main body of the systematic review using two or more electronic databases
- Description of the process for the selection of included studies (e.g. independent process, in duplicate, the number of authors involved)

### Search strategy

The search strategy was developed by one author (MC) and peer reviewed by an experienced Education and Health Sciences information specialist librarian (LD) using the Peer Review of Electronic Search Strategies checklist [57]. A three-step search strategy was utilised to ensure a comprehensive search of the literature [42]. The authors conducted an initial search limited to EMBASE and PubMed electronic databases to identify systematic reviews relevant to the overview research question. Initial key words were older adult, older person, senior, geriatric, emergency department, emergency care and systematic review. Subsequently, further key words within the titles and abstract were identified and analysed. Finally, index terms for the systematic reviews were analysed [42]. These steps guided the development of a comprehensive search strategy, which were adapted for each database.

The following electronic databases were searched during May 2020: the Cochrane Database of Systematic Reviews, Joanna Briggs Institute Database of Systematic Reviews and Implementation Reports, Databases of Abstracts of Reviews of Effects, PubMed 1966 to May 2020; OVID Medline 1996 to date; Embase 1974 to date; Cumulative Index to Nursing and Allied Health Literature (CINAHL) (EBSCO Host) 1981 to date; Epistemonikos; AGELINE 1978 to date; PEDro 1999 to date; Scopus and the PROSPERO register [43]. The search for unpublished systematic reviews and meta-analyses included OpenGrey, Google Scholar and MedNar. The final search strategies for eleven databases, the PROSPERO register and grey literature are detailed in Additional Information 1. Finally, the reference lists of all included systematic reviews were searched for additional relevant publications.

### Systematic review selection

#### Screening

Two independent reviewers (MC and RG) screened titles and abstracts in Endnote X8 (Clarivate Analytics, PA, USA) against the inclusion criteria for the umbrella review [42]. The authors of potentially relevant conference abstracts and protocols were contacted on three occasions to establish full text publication status. The full-texts of all potentially relevant systematic reviews were obtained and reviewed for eligibility by the same two independent reviewers (MC and RG). Additional information 2 details the list of excluded systematic reviews, conference abstracts and protocols. All relevant systematic reviews were screened for inclusion using the JBI Critical Appraisal Checklist for Systematic Reviews and Research Synthesis [42] (Additional Information 3). The authors (MC and RG) piloted this form on two systematic reviews to ensure consistency between reviewers. A score of 0–3 indicated a very low-quality score, thus the decision to include a review was made based on meeting a pre-determined proportion of > 3 of the 11 criteria [43, 53]. Comprehensive details of excluded systematic reviews following this assessment are presented in Additional Information 4. Any disagreements that arose between the authors were resolved through discussion.

#### Data extraction

Data were extracted from RCTs included in systematic reviews by one author (MC) and verified by another author (RG) using the standardised JBI data extraction form for systematic reviews and research syntheses [42] (Additional Information 5). The authors (MC and RG) piloted the form on two systematic reviews (a narrative synthesis and a meta-analysis) ensuring that the content and manner of data recording was accurate. In addition, the authors extracted data regarding the type of intervention and healthcare professionals involved in the intervention delivery. Where data discrepancies or omissions were suspected, the authors retrieved the data from seven RCTs that informed the systematic reviews [58–64] to ensure optimal accuracy and consistent data extraction [65].

#### Assessment of methodological quality

Methodological quality appraisal of the included systematic reviews was assessed by two independent reviewers (MC and RG) using the 16-item A MeaSurement Tool to Assess systematic Reviews 2 (AMSTAR 2) tool [66]. Three authors (MC, RG and SL) piloted the use of the AMSTAR 2 tool on two systematic reviews (a narrative synthesis and a meta-analysis) to ensure consistency between reviewers. Three authors (MC, RG and SL) identified and agreed on six key critical AMSTAR 2 items specific to the research question. Subsequently, the methodological quality of the systematic reviews were rated as high, moderate, low and critically low accordingly [66]. The rating was based on the following critical domains in the AMSTAR 2 tool [66] (to rate overall confidence in the results of the systematic review): Items 2,4,5,6,8,9.

#### Dealing with overlap

A list of the RCTs included in each included systematic review was collated and a matrix of evidence table was created and examined by two independent reviewers (MC and RG) to ascertain the degree of overlap between systematic reviews. Where there was 100% overlap in included RCTs across two or more systematic reviews, the AMSTAR 2 rating was used to decide which systematic review was retained. If AMSTAR 2 scores were equal, the most recently published systematic review was included. The authors (MC and RG) included all systematic reviews that analysed at least one additional RCT not in any other included systematic review in order to have the maximal amount of available data informing our outcomes.

#### Evaluation of the quality of the evidence

Two independent reviewers (MC and SL) applied an algorithm to assign Grading of Recommendations Assessment, Development and Evaluation (GRADE) approach to assess the strength of evidence for all outcomes for each systematic review [67]. In this algorithm, each systematic review started with a ranking of high certainty (no downgrade) and is downgraded one level per serious methodological concerns.

#### Data synthesis

Three authors (MC, RG and SL) analysed the data extracted to develop a narrative overview of the outcomes [42]. To facilitate comparison of intervention of effectiveness, we had planned to have a standardised approach to our results by converting the different estimates of effect that we extracted to one common effect measure [53]. However, these analyses were not possible due to the small number of meta-analyses and the heterogeneity between studies in terms of outcomes assessed. In light of the heterogeneity in populations, outcomes and analyses, the findings of included systematic reviews were summarised using a narrative synthesis with the quantitative tabulation of results as appropriate, as a meta-analysis was precluded.

Results were tabulated based on each outcome measure and presented as a narrative synthesis to address the research question of this umbrella review. The overall effect sizes and a description of the interventions are presented for interpretation of effectiveness of the interventions. The table of characteristics for included systematic reviews (Additional Information 6) includes extensive detail for each systematic review. The outcomes of each systematic review are considered based on the quality of the systematic review, as assessed by critical appraisal (AMSTAR 2) and algorithm to GRADE levels of evidence.

#### Deviations from the protocol

There were a number of challenges in the data extraction process in terms of varying information reported regarding the interventions. The authors (MC and RG) retrieved the primary RCTs that informed the systematic reviews to deal with this issue [65]. Data extraction was proposed to take place independently and in duplicate [53]. Data extraction for two systematic reviews were completed in this manner, but due to resource constraints data extraction for the following seven was carried out by one author (MC) and verified by second author (RG). The critical flaw domains of AMSTAR 2 were developed for this overview by the authors (MC and SL) following analysis of the included systematic reviews as recommended for overviews of specific healthcare interventions [66].

#### Patient and public involvement

Patients and the public were not involved in the conduct of this umbrella review. The findings of this umbrella review (which represents the development phase of the Medical Research Council framework for developing and evaluating complex interventions [68]) are anticipated to assist the design and development of a pilot feasibility intervention to address the effectiveness of interventions on the risk of adverse outcomes in older adults following discharge from the ED. The subsequent phases have a strong public and patient involvement, with a dedicated PPI panel of older adults established to support the researchers [69], including dissemination to academic and non-academic platforms.

## Results

### Study selection

**Fig. 1:**
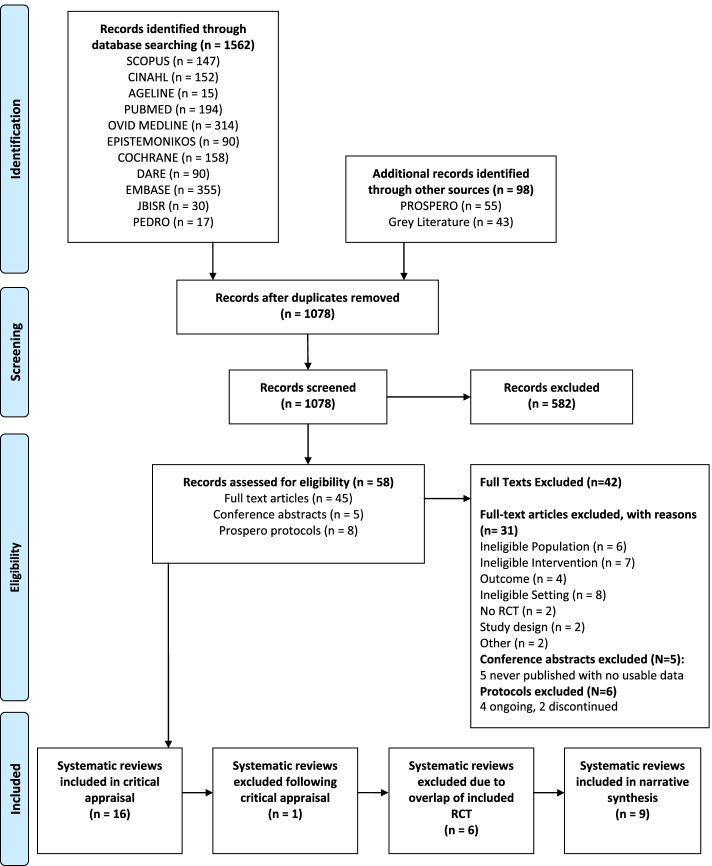
Umbrella Review Flow Diagram

Figure 1 summarises the study selection process. The literature search identified 1660 titles, of which 1562 were retrieved from 11 bibliometric databases. The search in grey literature and the PROSPERO databases identified 43 and 55 records respectively. Once duplicates were removed (*n* = 582) and title and abstracts were screened, a total of 58 records were deemed eligible for full-text review. Full-texts of five conference abstracts and six protocols registered with PROSPERO were not available; therefore 47 full texts were screened for eligibility. A total of 16 systematic reviews were assessed using the JBI Critical Appraisal Checklist for Systematic Reviews and Research Synthesis [42]. Additional Information 2 details the 31 excluded systematic reviews with reasons for exclusion.

The results of the JBI Critical Appraisal Checklist for Systematic Reviews and Research Synthesis [42], for each of the 16 systematic reviews are summarised in Table 1. The minimum number of criteria met was 3/11 [15] and the maximum was 10/11 [2, 36, 70]. One systematic review [15] was excluded on the basis of very low quality, with a critical appraisal score of 3 (Supplementary Information 4). Six systematic reviews were judged to be of low quality [19, 30, 37, 71, 72]; six were judged to be of moderate quality [16, 38, 39, 73–75] and three were deemed high quality [2, 36, 70]. Criteria 11, relating to specific directives for new research, was the only criteria to be met by all 16 systematic reviews.

**Table 1:** Critical Appraisal Results for Systematic Reviews using Joanna Briggs Institute Critical Appraisal Checklist for Systematic Reviews and Evidence Synthesis

	Aminzadeh & Dalziel 2002 [15]	Berning et al. 2020 [16]	Conroy et al. 2011 [38]	Fealy et al. 2009 [39]	Gates et al. 2008 [73]	Graf et al. 2011 [72]	Hastings & Heflin 2005 [30]	Hughes et al. 2019 [36]	Karam et al. 2015 [40]	Lowthian et al. 2015 [2]	Malik et al. 2018 [37]	McCusker & Verdon 2006 [19]	Morello et al. 2019 [74]	Pearce et al. 2011 [70]	Platzer et al. 2020 [71]	Pritchard et al. 2014 [75]
Item 1	U	Y	Y	U	Y	Y	Y	Y	Y	Y	Y	Y	Y	Y	Y	Y
Item 2	U	Y	Y	Y	Y	Y	Y	Y	Y	Y	Y	Y	Y	Y	Y	Y
Item 3	U	Y	U	U	Y	U	U	Y	Y	Y	U	U	Y	Y	U	Y
Item 4	Y	Y	Y	Y	Y	N	N	Y	Y	Y	Y	N	Y	Y	Y	Y
Item 5	U	Y	Y	U	Y	U	U	Y	U	Y	U	U	Y	Y	Y	Y
Item 6	U	Y	U	Y	U	U	U	Y	U	Y	U	U	U	Y	Y	U
Item 7	U	Y	U	Y	Y	U	U	U	U	Y	U	Y	Y	Y	N	Y
Item 8	U	U	Y	Y	Y	U	U	Y	U	Y	Y	Y	Y	Y	U	Y
Item 9	N/A	N/A	Y	N/A	U	N/A	N/A	Y	N/A	U	U	N/A	U	N/A	N/A	U
Item 10	Y	Y	Y	Y	Y	Y	Y	Y	Y	Y	Y	Y	Y	Y	U	Y
Item 11	Y	Y	Y	Y	Y	Y	Y	Y	Y	Y	Y	Y	Y	Y	Y	Y
Overall Score	**3**	**9**	**8**	**7**	**9**	**4**	**4**	**10**	**6**	**10**	**6**	**6**	**9**	**10**	**6**	**9**
Overall Quality	**VL**	**M**	**M**	**M**	**M**	**L**	**L**	**H**	**L**	**H**	**L**	**L**	**M**	**H**	**L**	**M**

*Y* Yes *N* No, *U* Unclear, *N/A* Non-applicable, *VL* Very low, *L* Low, *M* Moderate, *H* High

A score of 0–3 representing very low-quality score; a score of 4–6 represented a low-quality score; a score of 7–9 represented a moderate-quality score; and a score of 10–11 was considered a high-quality score

Item 1: Is the review question clearly and explicitly stated? Item 2: Were the inclusion criteria appropriate for the review question? Item 3: Was the search strategy appropriate? Item 4: Were the sources and resources used to search for studies adequate? Item 5: Were the criteria for appraising studies appropriate? Item 6: Was critical appraisal conducted by two or more reviewers independently? Item 7: Were there methods to minimize errors in data extraction? Item 8: Were the methods used to combine studies appropriate? Item 9: Was the likelihood of publication bias assessed? Item 10:

Were recommendations for policy and/or practice supported by the reported data? Item 11: Were the specific directives for new research appropriate?

### Assessment of methodological quality

Of the 15 systematic reviews that underwent quality appraisal assessment using the AMSTAR 2 tool [66], none were rated high quality. One systematic review and meta-analysis was rated as moderate quality [2], while the majority (*n* = 10) of the included systematic reviews were rated critically low [19, 30, 37–40, 71, 72, 74, 75]. Of note, four systematic reviews registered a protocol prior to commencement of the systematic review (Item 2) [2, 16, 36, 71], a critical domain within AMSTAR 2 quality rating [66]. Two systematic reviews [16, 36] were judged to have adequate detail to permit a “yes” answer to Item 4 relating to the conduct of a comprehensive search strategy. The majority of systematic reviews (*n* = 9) reported to have performed data extraction in duplication (Item 6), while only two [70, 73] provided justification for excluding individual studies (Item 7). Ten systematic reviews reported adequate detail in description of included studies for a “yes” on Item 8. No systematic review reported on the funding sources for the primary studies included in the systematic review. The full AMSTAR 2 results of all systematic reviews are available in Table 2 below.

**Table 2:** AMSTAR 2 results of eligible systematic reviews

	Berning et al. 2020 [16]	Conroy et al. 2011 [38]	Fealy et al. 2009 [39]	Gates et al. 2008 [73]	Graf et al. 2011 [72]	Hastings & Heflin 2005 [30]	Hughes et al. 2019 [36]	Karam et al. 2015 [40]	Lowthian et al. 2015 [2]	Malik et al. 2018 [37]	McCusker & Verdon 2006 [19]	Morello et al. 2019 [74]	Pearce et al. 2011 [70]	Platzer et al. 2020 [71]	Pritchard et al. 2014 [75]
Item 1	Y	N	Y	Y	Y	Y	Y	Y	Y	N	Y	N	Y	Y	Y
Item 2	Y	N	N	N	N	N	Y	N	Y	N	N	N	N	PY	N
Item 3	N	N	N	N	N	N	N	N	Y	N	Y	N	N	N	N
Item 4	Y	PY	PY	PY	N	PY	Y	PY	PY	PY	PY	PY	PY	PY	PY
Item 5	N	N	N	Y	N	Y	Y	Y	Y	Y	N	Y	Y	N	N
Item 6	Y	Y	Y	Y	N	N	N	N	Y	N	Y	Y	Y	N	Y
Item 7	N	N	N	Y	N	N	N	N	N	N	N	N	Y	N	N
Item 8	Y	PY	PY	Y	PY	Y	Y	Y	Y	Y	Y	PY	Y	PY	Y
Item 9	Y	PY	Y	Y	N	N	Y	N	Y	Y	N	Y	Y	PY	Y
Item 10	N	N	N	N	N	N	N	N	N	N	N	N	N	N	N
Item 11	N/A	Y	N/A	Y	N/A	N/A	Y	N/A	Y	Y	N/A	Y	N/A	N/A	Y
Item 12	N/A	Y	N/A	Y	N/A	N/A	Y	N/A	Y	N	N/A	Y	N/A	N/A	Y
Item 13	Y	Y	Y	Y	N	N	Y	N	Y	N	N	Y	Y	N	Y
Item 14	Y	Y	N	Y	N	N	Y	N	N	N	N	Y	Y	N	N
Item 15	N/A	Y	N/A	N	N/A	N/A	N	N/A	N	N	N/A	N	N/A	N/A	N
Item 16	Y	Y	Y	Y	Y	Y	Y	Y	Y	Y	Y	Y	Y	Y	Y
Overall Quality	**L**	**CL**	**CL**	**L**	**CL**	**CL**	**L**	**CL**	**M**	**CL**	**CL**	**CL**	**L**	**CL**	**CL**

*Y Yes*, *N* No, *PY* Partial Yes, *N/A* Non-applicable (no meta-analysis conducted), *CL* Critically low, *L* Low, *M* Moderate

Item 1: inclusion of PICO elements? Item 2: review methods established before conduct of review? Item 3: explanation for selection of study designs to be included in review? Item 4: use of a comprehensive search strategy? Item 5: selection of studies in duplicate? Item 6: data extraction in duplicate? Item 7: provision of list of excluded studies with justification for exclusion? Item 8: description of included studies in adequate detail? Item 9: satisfactory technique for risk of bias? Item 10: sources of funding for included studies reported? Item 11: proper methods for meta-analysis? Item 12: potential risk of bias in included studies discussed? Item 13: risk of bias accounted for in interpreting results? Item 14: heterogeneity discussed? Item 15: if meta-analysis conducted was publication bias discussed? Item 16: disclosure of funding or conflict of interest?

### Overlap

The 15 systematic reviews included 29 RCTs. Six systematic reviews were excluded [19, 70–73, 75] due to 100% overlap. The most commonly cited RCTs were Mion et al. 2003 [76], with 8 citations; Caplan et al., 2004 [77] with seven citations and Runciman et al., 1996 [78] with six citations. Table 3 illustrates the RCT citation count for each of the 15 systematic reviews, while Table 4 represents the final citation count for the nine systematic reviews included in data extraction following assessment of overlap and post exclusion of six systematic reviews.

**Table 3:** Matrix of Evidence (Citation) Table for 15 systematic reviews

CITED RCTs	SYSTEMATIC REVIEW CITATION															COUNT OF RCTs INCLUDED ACROSS SYSTEMATIC REVIEWS
	Berning et al. 2020 [16]	Conroy et al. 2011 [38]	Fealy et al. 2009 [39]	Gates et al. 2008 [73]	Graf et al. 2011 [72]	Hastings & Heflin 2005 [30]	Hughes et al. 2019 [36]	Karam et al. 2015 [40]	Lowthian et al. 2015 [2]	Malik et al. 2018 [37]	McCusker & Verdon 2006 [19]	Morello et al. 2019 [74]	Pearce et al. 2011 [70]	Platzer et al. 2010 [71]	Pritchard et al. 2014 [75]	
Gagnon et al. 1999	●		●		●	●	●			●	●					7
Joubert et al. 2013	●															1
McCusker et 2001	●		●		●	●	●		●							6
Mion et al. 2003	●	●	●		●	●	●	●	●	●	●					10
Runciman et al. 1996	●		●		●	●	●		●	●						7
Wilber et al. 2005	●												●			2
Davison et al. 2005		●		●								●				3
Caplan et al. 2004		●	●		●	●	●	●	●	●	●					9
McCusker et al. 2003a		●	●						●		●					4
Close et al. 1999		●	●	●								●				4
Basic & Conforti 2005			●		●		●			●						4
McCusker et al. 2003b			●						●		●					3
Lightbody et al. 2002				●								●				2
Shaw et al. 2003				●								●				2
Whitehead et al. 2003				●								●				2
Weir et al. 1998						●										1
Eklund et al. 2013							●									1
Biese et al. 2014							●									1
Biese et al. 2018							●									1
Lee et al. 2007								●								1
Yim et al. 2011									●							1
Cossette et al. 2015										●						1
Rosted et al. 2013										●						1
Barker et al. 2018												●				1
Chu et al. 2017												●				1
Harper et al. 2017												●				1
Hendricks et al. 2008												●		●	●	3
Matchar et al. 2017												●				1
Russell et al. 2010												●				1
Vind et al. 2009												●				1

*RCT* Randomised Controlled Trials

**Table 4:** Final Matrix of Evidence (Citation) Table for 9 Systematic Reviews included in Narrative synthesis

CITED RCTs	SYSTEMATIC REVIEW CITATION									COUNT OF RCTs INCLUDED ACROSS SYSTEMATIC REVIEWS
	Berning et al. 2020 [16]	Conroy et al. 2011 [38]	Fealy et al. 2009 [39]	Hastings & Heflin 2005 [30]	Hughes et al. 2019 [36]	Karam et al. 2015 [40]	Lowthian et al. 2015 [2]	Malik et al. 2018 [37]	Morello et al. 2019 [74]	
Gagnon et al. 1999	**●**		**●**	**●**	**●**			**●**		5
Joubert et al. 2013	●									1
McCusker et 2001	●		●	●	●		●			5
Mion et al. 2003	**●**	**●**	**●**	**●**	**●**	**●**	**●**	**●**		8
Runciman et al. 1996	**●**		**●**	**●**	**●**		**●**	**●**		6
Wilber et al. 2005	●									1
Davison et al. 2005		●							●	2
Caplan et al. 2004		**●**	**●**	**●**	**●**	**●**	**●**	**●**		7
McCusker et al. 2003a		●	●				●			3
Close et al. 1999		●	●						●	3
Basic & Conforti 2005			●		●			●		3
McCusker et al. 2003b			●				●			2
Lightbody et al. 2002									●	1
Shaw et al. 2003									●	1
Whitehead et al. 2003									●	1
Weir et al. 1998				●						1
Eklund et al. 2013					●					1
Biese et al. 2014					●					1
Biese et al. 2018					●					1
Lee et al. 2007						●				1
Cossette et al. 2015								●		1
Rosted et al. 2013								●		1
Barker et al. 2018									●	1
Chu et al. 2017									●	1
Harper et al. 2017									●	1
Hendricks et al. 2008									●	1
Matchar et al. 2017									●	1
Russell et al. 2010									●	1
Vind et al. 2009									●	1

*RCT* Randomised Controlled Trials

McCusker et al. 2001, 2003a and 2003b [61, 62] refer to the same RCT

### Description of the included systematic reviews

The nine included systematic reviews were conducted between 2005 and 2020 with the majority (*n* = 7) published after 2011. Pertinent details and characteristics from these systematic reviews are presented in Table 5 (Table of Characteristics). Full details of the characteristics of the included systematic reviews are available in Additional Information 6. Of the included systematic reviews, five performed meta-analysis [2, 36–38, 74] and four systematic reviews presented results of the included primary studies narratively [16, 30, 39, 40]. The four systematic reviews that were unable to conduct a meta-analysis reported that methodological and clinical heterogeneity observed in the RCTs precluded meta-analysis [16, 30, 39, 40].

**Table 5:** Summary Table of Included Nine Systematic Reviews

CITATION	NUMBER OF RCTs	DATE RANGE OF RCTs	PARTICIPANTS	INTERVENTION	OUTCOMES
Berning et al., 2020 [16] AMSTAR 2 rating: Low	6	1996 to 2013	1094	Interventions organised via themes "Care transitions" evaluating interventions involving care coordination within the ED and care related to post-ED discharge care coordination. Physical needs in the emergency care setting"	Patient experience or satisfaction
Conroy et al., 2011 [38] AMSTAR 2 rating: Critically Low	5	1999- 2005	2474	Comprehensive Geriatric Assessment (CGA)	Mortality Institutionalisation Functional outcomes: Barthel score Quality of Life: SF36 Cognition: Mini -Mental State Examination Readmissions: Full follow up period for all RCTS Readmission at 1 month
Fealy et al., 2009 [39] AMSTAR 2 rating: Critically Low	6	1996- 2005	2852	Gerontologically informed nursing assessment and referral intervention	Admission to hospital Length of stay Nursing home placement/admission Functional Decline Quality of Life: SF36 Patient and care giver satisfaction Readmission to ED
Hastings & Heflin 2005 [30] AMSTAR 2 rating: Critically Low	6	1999- 2004	NR	ED interventions (single and multi-strategy interventions)	Functional decline: (IADL and BADL, OARS), ED readmission, Institutionalisation Death
Hughes et al., 2019 [36] AMSTAR 2 rating: Low	9	1996- 2017	4561	ED interventions (single and multi-strategy interventions)	Functional decline ED readmission Patient experience Quality of Life Hospitalisation
Karam et al., 2015 [40] AMSTAR 2 rating: Critically Low	3	2003–2007	1475	CGA and PERS	ED revisits Hospital admission Mortality Nursing Home admission
Lowthian et al., 2015 [2] AMSTAR 2 rating: Moderate	5	1996 to 2011	3447	ED-based care transition	Functional decline in ADL Unplanned ED re-presentation: 1 month Emergency hospital admission: 1 month after initial attendance Mortality
Malik et al., 2018 [37] AMSTAR 2 rating: Critically Low	7	1996–2015	NR	Geriatric focused nurse assessment and interventions in the ED	Hospitalisation at day 30 post intervention Hospital readmission ED revisits
Morello et al., 2019 [74] AMSTAR 2 rating: Critically Low	12	1999–2018	3986	Multifactorial falls prevention interventions	Rate of falls: Falls calendars or diaries Number of fallers: Falls calendars or diaries Falls related ED presentation

*CGA* Comprehensive Geriatric Assessment

*ED* Emergency Department

*NR* Not reported

*PERS* Personal Emergency Response System

*RCT* Randomised Controlled Trial

### Search characteristics: databases

The minimum number of databases searched was two [30] and the maximum was eleven [38]. The databases searched most commonly were CINAHL [30, 36–40, 74], EMBASE [36–38, 40, 74], OVID Medline [16, 38, 74], MEDLINE [30, 37]. The search dates for the included systematic reviews ranged from 1996 to 2019 [16]. No search range was stated in one systematic review [40]. Five systematic reviews limited their searches to the English language [30, 37, 39, 40, 74]. The publication dates for the included RCTs ranged from 1996 to 2018. The number of RCTs included in each systematic review varied from three [40] to twelve [74]. The most cited country of origin of the RCTs were Australia (*N* = 8), USA (*N* = 5), UK (*N* = 5) and Canada (*N* = 5). The country of origin was not reported in one systematic review [38].

### Study and participant characteristics

The number of participants included in systematic reviews varied from 1094 [16] to 4561 [36]. Two systematic reviews did not report sample size [30, 37]. A minority of systematic reviews reported specific participant characteristics such as gender [36, 74] and co-morbidities [36]. All systematic reviews pertain to older adults although some systematic reviews included RCTs with participants over 65 years and over 75 years, both “high and low risk” patients presenting to the ED [36]. “High risk” patients were identified with risk screening profiling tools such as the Identification of Seniors at Risk tool, assessment of Activities of Daily Living [79], or by diagnosis. In general, the systematic reviews did not include a specific presenting condition or complaint. One systematic review included only frail older adults [38] while another systematic review focused on a sub-group of older adults; those who presented to the ED with a fall [74]. All systematic reviews included participants presenting to an ED in either a rural or an urban setting.

### Professional that carried out the intervention

The professionals that carried out the interventions included nurse case managers coordinating care [2, 16], nurses [16, 38, 39], community service providers [16], health visitor [16], medical social worker [80] and a geriatrician [36, 38]. A number of interventions involved an assessment and then referral on to community services providers.

### Critical appraisal of primary studies RCTs

The assessment of methodological quality of the included RCTs was based on different instruments, including Cochrane Collaboration Bias Appraisal tool [2, 16], Van Tulder scale [38]**,** a checklist described by Grimshaw et al. 2003 [39], Cochrane Effective Practice and Organisation of Care [36], RevMan 5.2 risk of bias tool [37] and the PEDro scale [74]. One systematic review with narrative synthesis did not report the name of the tool used to assess methodological quality [30], but did name the bias domains assessed. Another systematic review did not report any critical appraisal of included studies [40]. The systematic reviews that did perform critical appraisal were assessed to be of higher quality as per Criteria 5 on the JBI Critical Appraisal Checklist for Systematic Reviews and Research Synthesis than those that did not perform critical appraisal of included studies (Table 1). In addition to critical appraisal, two recent systematic reviews, one a narrative synthesis [16] and one incorporating a meta-analysis [36], utilised the Grading of Recommendations Assessment, Development and Evaluation (GRADE) approach to assess the certainty of evidence.

### Interventions

A variety of interventions are described in the nine systematic reviews. Eight systematic reviews focused on a specific intervention type. Two reviews focused on gerontologically informed nursing assessment and referral interventions [39, 40], and one each focused on comprehensive geriatric assessment (CGA) [38] and ED community transitional strategies [2]. One systematic review described the interventions as “ED based interventions” which included CGA in the ED with referral to community services; stratification of patients in the ED followed by referral to community services and the use of a Personal Emergency Response System plus a telephone call post discharge to assess ED outcomes [40]. One recent systematic review investigated both single strategy interventions (one intervention such as case management) and multi strategy interventions (more than one such as discharge planning, case management) [36]. One systematic review described a “variety of interventions” including CGA within the ED with referral to community services [30]. A recently published systematic review described the ED initiated interventions under a number of themes including “care transitions” and “physical needs in the emergency care setting” [16]. Specific details regarding the frequency, duration and intensity of the interventions were not reported in many of the systematic reviews [30, 37–40].

One systematic review focused solely on interventions to prevent falls in older adults presenting to the ED with a fall [74]. The risk assessment tools utilised and falls risk factors assessed varied substantially across RCTs. All 12 RCTs included in the review involved an assessment of falls risk factors. The falls risk factors assessed in the RCTs were home environment (10 RCTs), vision (10 RCTs), mobility or gait (nine RCTs) and balance (seven RCTs). The falls risk assessments were undertaken in a range of clinical settings and sometimes by more than one health care professional, including the patient’s home (10 RCTs), day hospital or clinic as an outpatient (four RCTs) or as an inpatient (two RCTs). The interventions described were very diverse, and included education (11 RCTs), a referral to other healthcare providers (11 RCTs), home assessments and adaptations (eight RCTs), exercise (six RCTs) and medication changes (5 RCTs). Some studies involved an option of treatments while other studies involved potential intervention strategies. The time from ED visit until the onset of the commencement of the intervention was reported in only six RCTs and varied from 2 to 8 weeks post baseline assessment. The frequency of the interventions varied from 1 to up to 16 sessions.

The comparator in the majority of nine systematic reviews was “usual care”. The components of usual care were not explored in the majority of systematic reviews [30, 37–40].

## Outcomes

### Primary outcomes

#### Clinical outcome: functional status/decline

Functional status or functional decline outcomes were reported narratively in five systematic reviews [2, 30, 36, 38, 39]. There was significant heterogeneity in the tools used to measure this outcome within the RCTs included in the five systematic reviews. These included changes in dependency in ADLs or IADLs; two RCTs reported change in functional status as a continuous outcome via Barthel Index (Caplan et al. 2004 [77]) and Older American Resources and Services Scale (OARS) tool (Gagnon et al. 1996 [81]). The quality of the systematic reviews reporting this outcome ranged from critically low [30, 38, 39] to moderate [2] quality (Table 2). Therefore there, is low quality evidence to support ED interventions in reducing functional decline, as presented in Table 6 below.

**Table 6:** Effectiveness of ED Interventions on functional status

OUTCOME	SYSTEMATIC REVIEW	OUTCOME MEASURE(S)	NUMBER OF RCTs INFORMING OUTCOME	NUMBER OF GRADE DOWNGRADES	GRADE LEVEL OF EVIDENCE
Functional Status/Decline	Conroy et al., 2011 [38]	Barthel score at 12 months	1	3	Low
	Fealy et al., 2009 [39]	Dependence in IADL and ADL at 4 weeks, ISAR tool, OARS	7	4	Low
	Hastings & Heflin 2005 [30]	Barthel score, IADL indices, OARS, Dependence in IADL and ADL at 4 weeks	4	5	Very low
	Hughes et al., 2019 [36]	Barthel score, IADL, OARS, Dependence in IADL and ADL at 4 weeks	5	3	Low
	Lowthian et al., 2015 [2]	Barthel score, IADL at 6 months	2	4	Low

*Abbreviations*: *ADL* Activities of Daily Living, *ED* Emergency Department, *IADL* Instrumental Activities of Daily Living, *RCT* Randomised controlled trial, *QOL* Quality of Life, *QARS* Older American Resources and Services Scale

### Quality of life

Three systematic reviews [30, 36, 38], ranging in quality from critically low to low on AMSTAR 2 rating (Table 2), investigated the effect of ED interventions on quality of life (QOL) of older adults discharged from the ED at index visit. One of the reviews was a systematic review with narrative synthesis [30] and two were systematic reviews with meta-analysis [36, 38]. The interventions described were CGA [38], ED interventions [30] and multi strategy interventions (discharge planning and case management) [36]. Three RCTs inform this outcome across the three systematic reviews with time periods to follow up of 30 and 120 days. The measurement tool used in the three RCTs was the Short Form-36 physical function and mental health component. There was no statistically significant effect of the ED intervention on either component of the SF36 at any time point, as presented in Table 7 below.

**Table 7:** Effectiveness of ED Interventions on Quality of Life

OUTCOME	SYSTEMATIC REVIEW	NUMBER OF RCT INFORMING OUTCOME	NUMBER OF DOWNGRADES	GRADE LEVEL OF EVIDENCE
Quality of Life	Conroy et al., 2011 [38]	1	3	Low
	Hastings & Heflin 2005 [30]	3	4	Low
	Hughes et al., 2019 [36]	2	3	Low

### Mortality

Four systematic reviews [2, 30, 38, 40], published from 2005 to 2019, investigated the effectiveness of ED interventions on mortality, as presented in Table 8 below. One systematic review and meta-analysis, of critically low quality as judged on AMSTAR 2 rating (Table 2), investigated the effectiveness of CGA [38], with five RCTs pooled informing this outcome, and time frame from one month to 18 months post ED index visit. There was no significant effect of CGA on mortality at final follow up. Two systematic reviews described the effectiveness of ED based interventions on mortality with the authors reporting no overall effect [30, 40]. Similarly, a moderate quality systematic review as judged on AMSTAR 2 rating (Table 2), reported that ED Community Transitional Strategies had no significant effect on mortality at 18 month follow up [2]. In total five RCTs informed this outcome across four systematic reviews. The quality of evidence across the four systematic reviews varied from very low to moderate.

**Table 8:** Effectiveness of ED Interventions on Mortality

OUTCOME	SYSTEMATIC REVIEW	NUMBER OF RCT INFORMING OUTCOME	NUMBER OF DOWNGRADES	GRADE LEVEL OF EVIDENCE
Mortality	Conroy et al. 2011 [38]	5	3	Low
	Hastings & Heflin 2005 [30]	3	5	Very Low
	Karam et al. 2015 [40]	2	4	Low
	Lowthian et al. 2015 [2]	2	2	Moderate

### Patient experience

Four systematic reviews investigated the effectiveness of ED interventions on patient experience or patient satisfaction [16, 30, 36, 39]. Seven RCTS in total contribute to this outcome across four systematic reviews. The most recently published systematic review focused on solely on this outcome reporting that improved patient experience was noted following department wide interventions [16]. There was significant heterogeneity in the in the tools used to measure patient experience and the methods of reporting this outcome [16]. The quality of the systematic review informing this outcome varied from critically low [39, 44] to low [16, 36] on AMSTAR 2 rating (Table 2). There is low quality evidence to support ED interventions in improving patient experience, as presented in Table 9 below.

**Table 9:** Effectiveness of ED Interventions on Patient experience or satisfaction

OUTCOME	SYSTEMATIC REVIEW	NUMBER OF RCT INFORMING OUTCOME	NUMBER OF DOWNGRADES	GRADE LEVEL OF EVIDENCE
Patient experience or satisfaction	Berning et al., 2020 [16]	6	3	Low
	Fealy et al., 2009 [39]	2	4	Low
	Hastings & Heflin 2005 [30]	4	4	low
	Hughes et al., 2019 [36]	4	3	Low

### Non-clinical outcome: Emergency department revisits/return visits

Emergency department (ED) return visit was an outcome in seven systematic reviews and systematic reviews with meta-analysis [38–40]**.** The majority of the systematic reviews informing this outcome are of a critically low quality [30, 37–40]. The quality of evidence of the effectiveness of ED interventions on reducing ED revisits varied from low to moderate. There were differences in the time period from index visit to ED return visit ranging from one month in one systematic review [2] to the end of follow up in another (up to 18 months) [39]. There was no effect of any ED intervention on ED return visits, as presented in Table 10 below.

**Table 10:** Effectiveness of ED Interventions on ED revisits/return visits

OUTCOME	SYSTEMATIC REVIEW	NUMBER OF RCT INFORMING OUTCOME	NUMBER OF GRADE DOWNGRADES	GRADE LEVEL OF EVIDENCE
Emergency department revisits	Conroy et al., 2011 [38]	5	3	Low
	Fealy et al., 2009 [39]	4	4	Low
	Karam et al., 2015 [40]	3	4	Low
	Hastings & Heflin 2005 [30]	4	4	Low
	Hughes et al., 2019 [36]	7	1	Moderate
	Lowthian et al., 2015 [2]	2	2	Moderate
	Malik et al. 2018 [37]	3	4	Low

### Hospital admissions

Five systematic reviews reported the effect of ED interventions on hospital admissions after the ED index visit [2, 30, 36, 37, 40]. ED readmissions was reported as a dichotomous outcome and a continuous outcome across the RCTs informing this outcome [36]. The time frames reported in the systematic reviews varied from 30 days [2, 37] to 60 days [40]. The quality of the systematic reviews informing this outcome ranged from critically low [30, 37, 40] to moderate quality [2]. There was no effect of any of the interventions described on hospital admissions after the ED index visit, as presented in Table 11.

**Table 11:** Effectiveness of ED Interventions on Hospital admissions

OUTCOME	SYSTEMATIC REVIEW	NUMBER OF RCT INFORMING OUTCOME	NUMBER OF DOWNGRADES	GRADE LEVEL OF EVIDENCE
Hospital admissions	Karam et al., 2015 [40]	3	4	Low
	Hastings & Heflin 2005 [30]	4	5	Very Low
	Hughes et al., 2019 [36]	5	2	Moderate
	Lowthian et al., 2015 [2]	2	2	Moderate
	Malik et al., 2018 [37]	3	4	Low

### Rate of falls

One systematic review with meta-analysis, of critically low quality, investigated the effectiveness of multifactorial falls ED interventions on rate of falls [74]. Nine RCTs informed this outcome and the overall quality of the evidence was low. The meta-analysis reported that multifactorial ED interventions did not reduce falls, as presented in Table 12 below.

**Table 12:** Effectiveness of ED Interventions on Rate of falls

OUTCOME	SYSTEMATIC REVIEW	NUMBER OF RCT INFORMING OUTCOME	NUMBER OF DOWNGRADES	GRADE LEVEL OF EVIDENCE
Rate of falls	Morello et al. 2019 [74]	9	4	Low

### Number of fallers

One systematic review with meta-analysis reported the effectiveness of multifactorial ED interventions on number of fallers with time frames varying from 6 to 12 months follow up [74], as presented in Table 13. There was moderate quality evidence for reducing the number of fallers in this systematic review, which was of critically low quality.

**Table 13:** Effectiveness of ED Interventions on Number of fallers

OUTCOME	SYSTEMATIC REVIEW	NUMBER OF RCT INFORMING OUTCOME	NUMBER OF DOWNGRADES	GRADE LEVEL OF EVIDENCE
Number of fallers	Morello et al. 2019 [74]	12	2	Moderate

No systematic review reported the outcome of length of ED stay.

## Discussion

### Main findings

This comprehensive umbrella review included nine systematic reviews representing 29 RCTs investigating the effectiveness of ED interventions to reduce adverse outcomes amongst older adults following discharge from the ED. We identified low quality evidence for the effectiveness of interventions for outcomes such as functional decline, patient experience, quality of life, mortality and rate of falls. There was variable quality of evidence, from low to moderate, for the effectiveness of interventions aiming to reduce health care utilisation (ED return visits and hospital admissions). There was moderate quality of evidence for ED interventions at reducing the number of fallers. This umbrella review highlights the challenge of synthesising the results of each outcome from each systematic review as there was significant heterogeneity including methodological heterogeneity of the conduct of the reviews (critical appraisal tools used), heterogeneity of the descriptions of the interventions within the RCTs, and the reporting of the outcomes. It is difficult to draw robust and definite conclusions from the findings of each systematic review and thus synthesise. We have highlighted gaps and recommendations for research and practice with respect to relevant ED guidelines and research priorities for geriatric emergency medicine [82, 83].

### Methodological issues of systematic reviews

Within the eight systematic reviews that focused on a specific intervention type [2, 16, 30, 36–40], there was substantial clinical heterogeneity and heterogeneity in the reporting of the patient characteristics including the age profile, gender and presence of co-morbidities. Given that age itself is cited as a risk factor for negative health outcomes [15], it is difficult to compare across age profiles. There was a variety of different descriptions and labelling of the interventions. The interventions were referred to as comprehensive geriatric assessment, gerontological nursing interventions, ED-Community transitional strategies but the same RCTs informed the outcomes of the systematic reviews. The matrix of evidence table indicates the degree of overlap within the systematic reviews. Within the RCTs, while there was a lack of in-depth descriptions of the interventions [36], there was a form of geriatric assessment plus referral to support services within the community setting. Given the complexity of the setting and the population group, precision for defining intervention is imperative [84]. Furthermore, there was a lack of information regarding the duration, frequency of the interventions and the precise healthcare professional delivering specific components of the interventions and a description of usual care was not consistency reported in RCTs. One author of an included systematic review reported difficulty in extracting data due to inconsistencies in reporting results [40]. There is a clear need for intervention fidelity to assess the reliability and validity of the interventions [16]. Within the one systematic review focused solely on interventions to prevent falls in older adults presenting to the ED with a fall [74], the interventions delivered were generic interventions based on interventions delivered to community dwelling older adults and not specific to older adults presenting to the ED. As older adults presenting to the ED as a result of a fall are often older, they are at a higher risk of future falls and present with multimorbidity [15, 54], thus pose a different clinical challenge and a more bespoke intervention. In considering the evidence reported in this umbrella review there is a need to consider the setting of the ED itself as a source of heterogeneity in terms of capacity, policy and procedures of the interventions. The context setting [85] of the majority of the RCTs were conducted in Europe, Australia and Canada and date back to 1996.

An umbrella review is limited by what authors of systematic reviews have already analysed and synthesised [86] and the RCTs that they include [16, 44, 85]. In this umbrella review, there was significant overlap between the systematic review with essentially five RCTS, dating from to 1996 to 2004 informing outcomes such as functional decline, ED revisits and ED admissions after the index visit, rendering the evidence base remarkably limited. A further challenge to comparing evidence across systematic reviews is the different tools used for critical appraisal of the RCTs included in the systematic review [44]. The relationship between the methodological quality of the systematic review and the conclusions of the systematic review have not yet been established [44].

There have been no systematic reviews published that focus on recommendations from the Emergency Medicine and Geriatric organisations in the UK and USA [25]. The 2014 Emergency Medicine Guidelines present recommendations in developing a “geriatric friendly” ED. The recommendations include issues pertaining to administration, physical environment and staffing.

### Outcomes

The number of outcomes reported in the included systematic reviews were a combination of patient and process outcomes. The diversity of outcome measures presents a challenge to determine the effectiveness of any intervention or interventions.

### Functional status

An important relationship exists between function and adverse outcomes in older adults [87] and functional assessment with validated tools is an important component of an ED assessment of an older adult [23]. A systematic review evaluating the use of functional assessments utilised in the ED identified 14 such assessments, but only four assessments were developed for use in the ED [23]. The authors of this systematic review reported that the assessments were always self-administered using self-report rather than patient observation. The authors reported limited psychometric testing has been completed on functional assessments within the ED. This observation is reflected in the findings of this umbrella review with multiple assessment tools utilised to assess functional decline rendering comparisons across systematic reviews difficult.

### Healthcare utilisation: ED revisits and hospital admission

There was substantial differences in the time period from index visit to ED return visit ranging from one month in one systematic review [2] to the end of follow up in another (up to 18 months) [39].The optimal time to determine significant changes in healthcare utilisation outcomes is currently unknown [36]. Methodological issues within the RCTs challenge comparisons with some RCTS reporting ED return visit as a dichotomous outcome and others as a continuous outcome. There is a need for consistency or uniformity in the assessment of this outcome in order to allow pooling of data. The utility of this outcome as a measure of effectiveness of ED interventions is questionable as the decision to attend an ED or not may not be related to the intervention of healthcare professional delivering the intervention [39]**.** Rather**,** increased service use may be a positive impact as it may lead to healthcare monitoring and health promotion [39]. Recent commentary on research priorities for geriatric emergency medicine question the use of process outcomes (LOS, ED revisit, unplanned hospital admission) as there is uncertainty uncertain to their association with patient well-being [82]. Older adults are vulnerable to the negative consequences of hospital admission [88] and ED interventions are often employed to reduce this form of healthcare utilisation. Similar to ED revisits, this form of healthcare utilisation may reflect improved healthcare monitoring of an at risk population [2]. Other factors such as past use of healthcare services and satisfaction with a service are important variables influencing healthcare utilisation [89].

### Patient experience

There was significant heterogeneity in the assessment tools used to ascertain patient experience or satisfaction and none of the RCTS had validated the tools in the ED setting [16]. Many of the RCTs used satisfaction-based questionnaires as a measurement tool of patient experience**.** A limitation of satisfaction-based questionnaires is the potential for gratitude bias [90]. One systematic review author called for an emphasis on the experience of the service and whether the experience can be improved [16]. The emphasis on the experience of the service is also identified within this umbrella review is also echoed in a qualitative meta-analysis of 22 studies exploring patient experience in all age groups in the ED [91].

### Quality of life

Quality of life is associated with fear of falling and subjective well-being in older adults presenting to the ED [92], however the effectiveness of ED interventions on quality of life of older adults was explored in only three RCTs informing three systematic reviews. The periods to follow up ranged between 30 and 120 days, thus comparisons are challenging. Given the very limited investigation of this outcome across RCTs, further research is warranted and this is a key research priority for geriatric medicine [82].

### Implications for practice

There is low quality evidence for the effectiveness of ED intervention for older adults post ED discharge for patient centred outcomes. The authors of all systematic reviews included in this umbrella review recommend that more high quality RCTs need to be conducted in this area. Other recommendations include research into interventions that bridge the ED- community transition [38]. One systematic review author suggested the use of a pragmatic trial [39] given the function of ED for service provision, while a hybrid mixed methods design was also suggested [2]. The 2014 Geriatric ED guidelines were developed to enhance the care of older adults,and while these guidelines are based on evidence from studies in the inpatient and outpatient clinical settings and not the ED setting [93], it seems pragmatic to follow these guidelines in conducting trials.

The authors of this umbrella review did not identify any systematic review that explored an ED intervention targeting older adults led by allied health and social care professionals (HSCPs) such as physiotherapists and occupational therapists. Research has demonstrated that HSCPs can have a positive impact in the ED in improving patient experience, reducing length of ED stay and preventing hospital admissions in other age groups [94–97]. Given the fact that CGA is delivered by multiple disciplines in other care settings [29], it would seem pragmatic to deliver the same in the ED. Delivery of a geriatric medicine intervention requires a whole systems approach with multiple health care professionals [83]. The Acute Frailty network in the UK is an example of a whole systems approach for older people admitted to hospital and has shown improvement in patient outcomes [98]. A research priority identified by the European Society for Emergency Medicine Geriatric Emergency Medicine [4] section and the European Geriatric Medicine Society GEM Special Interest Group is to identify if elements of CGA are effective in improving outcomes for older patients [83]. Other key research questions included what community interventions are safe and effective to prevent adverse outcomes for older adults following discharge from the ED.

The ED has been termed a “front porch” prior to discharge home into the community [83, 98–100] thus it is a high research priority to further investigate the effectiveness of an ED community transitional strategy to improve outcomes in older adults discharged from the ED[101]. An evidence based synthesis of ED intervention for older adults conducted for the Department of Veteran Affairs recommended working across clinical settings and healthcare disciplines [102]. There has been a call for innovation with patient centred programs [99].

A umbrella review [48] summarised which ED interventions met the needs of older adults and reported no individual intervention was more beneficial. This umbrella review focused on the interventions delivered within the ED and in-patient settings, while our umbrella review explored the interventions based in the ED, or initiated in the ED, and transitional interventions as well as the outcomes reported in systematic reviews. We agree with the findings of this umbrella review and in our umbrella review, we applied an algorithm to GRADE to assess the strength of evidence for all outcomes in each systematic review thus providing a comprehensive summary of evidence in this field. The degree of overlap was taken into account and presented in detailed matrix of evidence tables. This review of reviews also recommended RCTs to focus on patient centred outcomes [48].

Our results are in line with other umbrella reviews investigating interventions to improve hospital admissions and transitional care strategies in adult populations in calling for more robust RCTS that extend beyond the hospital stay and meet the patients’ needs [103, 104]. A systematic review investigating the effect of transitional care interventions on hospital readmissions in older medical patients also reported that more RCTs are required that include an intervention with minimum duration of one month and that target high- risk patients [105].

### Implications for research

Authors of systematic reviews have called for a larger set of core outcome measures that encompass both patient and service priorities [16, 36]. The investigation of individually tailored multi-strategy interventions and integrated care are further avenues for research [106]. In line with general recommendations for the care of older adults in all settings, the identification of patient preferences is imperative [21, 106]. A clear understanding of the unique needs of older adults [21, 107, 108] with the incorporation of feedback from patients [6, 36] and key stakeholders has the potential to deliver an intervention that is acceptable to patients and service providers [16, 106]. An investigation of the experiences of older adults in ED using ED validated tools of patient experience is warranted [16]. There is a need for the engagement with patients and stakeholders to define and identify appropriate outcome measures and align these with clinical outcomes. These recommendations are in line with ED GEM research priorities to consult with patients prior to the implementation of an intervention [83].

Most of the included systematic reviews included in this umbrella review did not report how the ED interventions were designed and developed, which is also the case for in-hospital interventions for reducing readmissions to the ED and acute care services for older adults [88]. However, given the efficacy of CGA in other care settings [29], it would seem pragmatic to implement elements of CGA such as screening for frailty in the ED.

### Strengths of this umbrella review

There are several strengths of this umbrella review especially the use of the JBI methodology and an a priori peer reviewed protocol published to guide the conduct of this umbrella review. A comprehensive search of the literature which included both published and unpublished sources of information with no limitations on language and publication date is another strength of this umbrella review. The use of the AMSTAR 2 for assessment of methodological quality as well as an algorithm to GRADE to assess the strength of evidence add to the robustness.

### Limitations

Although the literature search attempted to locate unpublished research, only one unpublished systematic review was identified. The degree of overlap was extensive also. Efforts to minimise overlap were dealt with according to current best practice [65]. Additionally, there are no specific reporting guidelines for umbrella reviews of healthcare interventions although research has commenced in this area [52].

## Conclusions

Rising ED visits and an ageing population with chronic health issues render ED interventions to reduce adverse outcomes in older adults a research priority. The existing evidence base for the effectiveness of ED interventions in reducing adverse outcomes is limited, and this limited evidence base is due to the poor quality of the RCTs. Higher quality intervention RCTs as well as a focus on intervention development with the engagement of stakeholders are required.

## Supplementary Information

**Additional file 1:** **Supplementary Information 1.** Search Strategy.

**Additional file 2:** **Supplementary Information 2.** List of Excluded full text systematic reviews, Protocols registered on PROSPERO, and Conference Abstracts.

**Additional file 3:** **Supplementary Information 3. **Joanna Briggs Institute Critical Appraisal Checklist for Systematic Reviews.

**Additional file 4:** **Supplementary Information 4.** List of Excluded Full text systematic reviews following assessment with Joanna Briggs Institute Critical Appraisal Checklist for Systematic Reviews.

**Additional file 5:** **Supplementary Information 5.** Data Extraction form.

**Additional file 6:** **Supplementary Information 6.** Table of Characteristics of 9 included systematic reviews.

**Additional file 7:** **Supplementary Information 7.** Algorithm to Grading of Recommendations, Assessment, Development and Evaluation (GRADE) for all for all outcomes in each systematic review.

## Data Availability

All data generated or analysed during this study are included in this published article and its supplementary information files.

## Abbreviations

ADL: Activities of Daily Living
AMSTAR 2: A MeaSurement Tool to Assess systematic Reviews 2 (tool)
AMU: Acute Medical Unit
CGA: Comprehensive Geriatric Assessment
ED: Emergency Department
GEM: Geriatric Emergency Medicine
GRADE: Grading of Recommendations Assessment, Development and Evaluation
JBI: Joanna Briggs Institute
IADL: Instrumental Activities of Daily Living
LOS: Length of stay
OARS: Older American Resources and Services Scale
PICOS: Population, Intervention, Comparator, Outcome and Study design
PRIOR: Preferred Reporting Items for Overviews of Reviews
RCTs: Randomised controlled trials
