# Supplementary material for: The effectiveness of interventions to reduce adverse outcomes among older adults following Emergency Department discharge: umbrella review
Source: BMC Geriatr. 2022 May 28;22:462. doi: 10.1186/s12877-022-03007-5 (PMC9145107; doi:10.1186/s12877-022-03007-5)
Supplement: Supplementary file 1 — Additional file 1: Supplementary Information 1. Search Strategy. [file 12877_2022_3007_MOESM1_ESM.docx]

**Supplementary Information 1: Search Strategy**

Search date: Up to June 2020

Search history sorted by search number ascending

**COCHRANE DATABASE OF SYSTEMATIC REVIEWS**

| **SEARCH NUMBER** | **QUERY** |
| --- | --- |
| #1 | (older person* OR older adult* OR aged OR geriatric* OR geriatric assessment OR aged, 80 and over OR senior* OR elderly OR old OR older OR frail elderly):ti,ab,kw (Word variations have been searched) |
| #2 | MeSH descriptor: [Aged] explode all trees |
| #3 | MeSH descriptor: [Frail Elderly] explode all trees |
| #4 | MeSH descriptor: [Geriatric Assessment] this term only |
| #5 | #1 OR #2 OR #3 OR #4 |
| #6 | (emergency service, hospital OR emergency medical services OR emergency near/2 service OR emergency medicine OR emergency department OR emergency care OR urgent care):ti,ab,kw (Word variations have been searched) |
| #7 | MeSH descriptor: [Emergency Service, Hospital] explode all trees |
| #8 | MeSH descriptor: [Emergency Medicine] this term only |
| #9 | #6 OR #7 OR #8 |
| #10 | (systematic review):ti,ab,kw (Word variations have been searched) |
| #11 | #5 AND #9 AND #10 |

**EMBASE (Elsevier)**

| **SEARCH NUMBER** | **QUERY** |
| --- | --- |
| #1 | 'aged'/exp |
| #2 | 'older adults':ti,ab OR 'older adult':ti,ab OR 'older people':ti,ab OR 'older patient':ab,ti OR 'older patients':ab,ti OR 'very elderly' OR senior:ti,ab OR seniors:ab,ti OR 'aged':ti,ab OR 'geriatric patient':ab,ti OR 'geriatric care'/exp OR 'geriatric care' OR 'geriatrics'/exp OR 'geriatrics' OR geriatric:ti,ab OR 'geriatric assessment'/exp OR 'geriatric assessment' OR 'gerontology':ab,ti |
| #3 | 'very elderly'/exp OR 'very elderly' |
| #4 | “elderly care”/exp |
| #5 | #1 OR #2 OR #3 OR #4 |
| #6 | 'emergency health service'/exp OR 'hospital emergency service'/exp |
| #7 | 'emergency ward'/exp |
| #8 | 'emergency department':ab,ti OR 'emergency departments':ab,ti OR 'emergency ward':ab,ti OR 'emergency treatment':ab,ti OR 'emergency health service' OR 'emergency room':ab,ti OR 'hospital':ab,ti OR 'emergency unit':ab,ti OR 'trauma unit':ab,ti OR 'emergency nursing':ab,ti OR 'emergency care':ti,ab OR 'acute medical unit':ab,ti OR 'emergency medicine':ab,ti OR (emergency NEAR/3 service) |
| #9 | #6 OR #7 OR #8 |
| #10 | 'systematic review':ab,ti |
| #11 | #5 AND #9 AND #10 |

**JOANNA BRIGGS INSTITUTE (JBI) EVIDENCE BASED PRACTICE DATABASE (formerly JBI DATABASE OF SYSTEMATIC REVIEWS AND IMPLEMENTATION REPORTS)**

| **SEARCH NUMBER** | **QUERY** |
| --- | --- |
| #1 | aged OR older OR elder* |
| #2 | 'older adults':ti,ab OR 'older adult':ti,ab OR 'older people':ti,ab OR 'older patient':ab,ti OR 'older patients':ab,ti OR aged :ti,ab |
| #3 | (geriatric* or elderly). |
| #4 | #1 OR #2 OR #3 |
| #5 | 'emergency department':ab,ti OR 'emergency departments':ab,ti OR 'emergency ward':ab,ti OR 'emergency treatment':ab,ti |
| #6 | Emergency* |
| #7 | #5 OR #6 |
| #8 | #4 AND #7 |

**OVID MEDLINE(R) and Epub Ahead of Print, In-Process & Other Non-Indexed Citations, Daily and Versions(R)**

| **SEARCH NUMBER** | **QUERY** |
| --- | --- |
| #1 | exp Aged/ |
| #2 | exp Geriatrics/ |
| #3 | exp "Aged, 80 and over"/ |
| #4 | exp Frail Elderly/ |
| #5 | (((older person* OR older adult* OR aged OR geriatric* OR geriatric assessment or aged, 80) and over) OR senior* OR elderly OR old OR older or frail elderly).ab. OR (((older person* OR older adult* OR aged OR geriatric* OR geriatric assessment OR aged, 80) and over) OR senior* OR elderly OR old OR older OR frail elderly).ti. |
| #6 | exp Health Services for the Aged/ |
| #7 | #1 OR #2 OR #3 OR #4 OR #5 OR #6 |
| #8 | exp Emergency Service, Hospital/ |
| #9 | exp Emergency Medical Services/ |
| #10 | emergency [medicine.mp](http://medicine.mp/). OR exp Emergency Medicine/ |
| #11 | 'emergency department':ab,ti OR 'emergency departments':ab,ti OR 'emergency ward':ab,ti OR 'emergency treatment':ab,ti |
| #12 | ("accident and emergency" OR "A&E" OR "A and E").tw. |
| #13 | ((emergency service, hospital or emergency medical services OR emergency) adj2 service) OR emergency medicine OR emergency department OR emergency care OR urgent care).ab. OR (((emergency service, hospital OR emergency medical services or emergency) adj2 service) OR emergency medicine OR emergency department OR emergency care OR urgent care).ti. |
| #14 | #8 OR #9 OR #10 OR #11 OR #12 OR #13 |
| #15 | systematic review.ab. OR systematic review.ti. |
| #16 | #7 AND #14 AND #15 |

**PUBMED (WWW.PUBMED.GOV)**

| **SEARCH NUMBER** | **QUERY** |
| --- | --- |
| #1 | "Aged"[MH] |
| #2 | (("AGED, 80 AND OVER"[MH] |
| #3 | "Aging"[MH] |
| #4 | "Geriatric Assessment"[MH] |
| #5 | "Geriatrics"[MH] OR “Geriatricians”[MH] |
| #6 | "Health Services for the Aged"[MH] |
| #7 | ("older adults"[Title/Abstract] OR "older people"[Title/Abstract] OR "older patients"[Title/Abstract] OR "elder*"[Title/Abstract] OR "elders"[Title/Abstract] OR "elderly"[Title/Abstract] OR "frail elderly"[Title/Abstract] OR "geriatric"[Title/Abstract] OR “aging*” OR "geriatrics"[Title/Abstract]) |
| #8 | #1 OR #2 OR #3 OR #4 OR #5 OR #6 OR #7 |
| #9 | “Emergency Medical Services”[MH] |
| #10 | "Emergency Medicine"[MH] |
| #11 | "Emergency service, Hospital"[MH] |
| #12 | “Emergency Treatment"[MH] |
| #13 | “Emergency Nursing"[MH] |
| #14 | ("emergency department"[Title/Abstract] OR "emergency room"[Title/Abstract] OR "emergency unit"[Title/Abstract] OR “emergenc*"[Title/Abstract] OR "emergency ward"[Title/Abstract] OR "Trauma unit"[Title/Abstract] OR "urgent care"[Title/Abstract] OR "emergency units"[Title/Abstract] OR "trauma unit"[Title/Abstract] OR "trauma units"[Title/Abstract])) OR ("trauma centers"[Title/Abstract] OR "trauma center"[Title/Abstract]) |
| #15 | #9 OR #10 OR #11 OR #12 OR #13 OR #14 |
| #16 | #8 AND #15 |
| #17 | 'systematic review': Filter publication type |

**CINAHL**

| **SEARCH NUMBER** | **QUERY** |
| --- | --- |
| S1 | MH "Aged" |
| S2 | MH "Frail Elderly" |
| S3 | MH "Aged, 80 and Over" |
| S4 | MH ("Geriatric Assessment") OR MH “Geriatrics” |
| S5 | MH "Aging” |
| S6 | TI (“older adults) |
| S7 | TI ((older N2 (patient* or adult* or people or person*)) OR AB ((older N2 (patient* or adult* or people or person*)) |
| S8 | TI (frail or geriatric* or elderly) OR AB (frail or geriatric* or elderly) |
| S9 | S1 OR S2 OR S3 OR S4 OR S5 OR S6 OR S7 OR S8 |
| S10 | (MH "Emergency Medical Services") OR (MH "Emergency Service") |
| S11 | TI ((emergency N2 (unit or units or room or rooms or ward or wards or department* or service*)) OR AB ((emergency N2 (unit or units or room or rooms or ward or wards or department* or service*)) |
| S12 | TI ("accident and emergency" or “Emergency Department” or "A&E" or "ED" or "A and E" ) OR AB ("accident and emergency" or “Emergency Department” or "A&E" or "ED" or "A and E") |
| S13 | S10 OR S11 OR S12 |
| S14 | TI “systematic review” OR AB “systematic review” |
| S15 | S9 AND S13 AND S14 |

**PROSPERO REGISTER**

| **SEARCH NUMBER** | **QUERY** |
| --- | --- |
| #1 | MeSH DESCRIPTOR Aged EXPLODE ALL TREES |
| #2 | MeSH DESCRIPTOR Aged 80 and over EXPLODE ALL TREES |
| #3 | MeSH DESCRIPTOR Health Services for the Aged EXPLODE ALL TREES |
| #4 | "older adult" OR "older people" OR “elderly” OR “senior” OR “elder” OR “senior” OR “elderly” OR “old people” |
| #5 | #1 OR #2 OR #3 OR #4 |
| #6 | MeSH DESCRIPTOR Emergency Medical Services EXPLODE ALL TREES |
| #7 | MeSH DESCRIPTOR Emergency Service, Hospital EXPLODE ALL TREES |
| #8 | MeSH DESCRIPTOR Emergency Medicine EXPLODE ALL TREES |
| #9 | “emergency department” OR “emergency room” OR “emergency unit” OR “trauma unit” |
| #10 | #6 OR #7 OR #8 OR #9 |

**EPISTEMEMONIKOS**

| **SEARCH NUMBER** | **QUERY** |
| --- | --- |
|  | (title:(“older adults” OR "older people" OR “elderly” OR “senior” OR “elder” OR” OR “old people”) OR (abstract:(“older adults” OR "older people" OR “elderly” OR “senior” OR “elder” OR” OR “old people”)) AND (title:(“emergency department” OR “emergency room” OR “emergency unit” OR “emergency ward” OR “trauma unit”) OR abstract:(“emergency department” OR “emergency room” OR “emergency unit” OR “emergency ward” OR “trauma unit”)) |

**AGELINE (EBESCO)**

| **SEARCH NUMBER** | **QUERY** |
| --- | --- |
| #1 | 'emergency department':ab,ti OR 'emergency departments':ab,ti OR 'emergency ward':ab,ti OR 'emergency treatment':ab,ti OR emergenc* |
| #2 | “systematic review” :ab,ti |
| #3 | #1 AND #2 |
| #4 | Limiter: academic journals |

**SCOPUS**

| **SEARCH NUMBER** | **QUERY** |
| --- | --- |
| #1 | TITLE-ABS-KEY ("aged” OR elder* OR “old people” OR geriatric* OR “oldest old” OR senior* OR “senium” OR “very old” OR "geriatrics" OR “older adult” OR “older adults” OR “older people” OR “frail*) |
| #2 | TITLE-ABS-KEY ("Geriatric Assessment") OR (“Geriatric assessments”) |
| #3 | #1 OR #2 |
| #4 | TITLE-ABS-KEY (“Emergency Medical Services”) |
| #5 | TITLE-ABS-KEY (“Emergency Hospital Services”) |
| #6 | TITLE-ABS-KEY (Emergency Service”) |
| #7 | TITLE-ABS-KEY (Emergency Services”) |
| #8 | TITLE-ABS-KEY (Emergency Health Service”) |
| #9 | TITLE-ABS-KEY (Emergency Health Service”) |
| #10 | TITLE-ABS-KEY (“emergency room” OR “emergency department' or “emergency medicine” OR “emergency medicine" OR "emergency department" OR "emergency care" OR "urgent care”) |
| #11 | #4 OR #5 OR #6 OR #7 OR #8 OR #9 OR #10 |
| #12 | TITLE-ABS-KEY (“systematic review”) |
| #13 | #3 AND #11 AND #12 |

**DATABASE OF REVIEWS OF EFFECTS (DARE)**

| **SEARCH NUMBER** | **QUERY** |
| --- | --- |
| S1 | old* (AB) |
| S2 | elder* (AB) |
| S3 | geriatric* |
| S4 | emergency department (AB) |
| S5 | MeSH DESCRIPTOR Frail Elderly EXPLODE ALL TREES |
| S6 | TI ((emergency N2 (unit or units or room or rooms or ward or wards or department* or service*)) OR AB ((emergency N2 (unit or units or room or rooms or ward or wards or department* or service*)) |
| S7 | MH ("Geriatric Assessment") |
| S12 | (MH "Emergency Medical Services") OR (MH "Emergency Service") |

**PEDro PHYSIOTHERAPY EVIDENCE DATABASE**

"older adults" age* emergenc* “systematic review”

**GOOGLE SCHOLAR, MEDNAR AND OPEN GREY LITERATURE**

| **SEARCH NUMBER** | **QUERY** |
| --- | --- |
| S1 | older  aged  frail  elder  emergency department  geriatric medicine  review  (With all of the words) |
| S2 | Aged OR  Elder OR  Frail OR  Emergency department  Emergency medicine  Accident and emergency  Systematic review |
| S3 | #1 AND #2 |
