# Supplementary material for: The effectiveness of interventions to reduce adverse outcomes among older adults following Emergency Department discharge: umbrella review
Source: BMC Geriatr. 2022 May 28;22:462. doi: 10.1186/s12877-022-03007-5 (PMC9145107; doi:10.1186/s12877-022-03007-5)
Supplement: Supplementary file 2 — Additional file 2: Supplementary Information 2. List of Excluded full text systematic reviews, Protocols registered on PROSPERO, and Conference Abstracts. [file 12877_2022_3007_MOESM2_ESM.docx]

**Supplementary Information 2: List of Excluded full text systematic reviews, Protocols registered on PROSPERO, and Conference Abstracts**.

**(a)**

**LIST OF EXLUDED FULL TEXT SYSTEMATIC REVIEWS WITH REASONS FOR EXCLUSION**

| **AUTHORS AND YEAR** | **TITLE** | **REASON FOR EXCLUSION *USING PICO FRAMEWORK*** |
| --- | --- | --- |
| Dickens, C.  Katon, W.  Blakemore, A.  Khara, A.  Tomenson, B.  Woodcock, A.  Fryer, A.  Guthrie, E.  Year: 2014 | Complex interventions that reduce urgent  care use in COPD: A systematic review with  meta-regression | Ineligible population:  Adult not “older adults “ |
| Echevarria, C.  Brewin, K.  Horobin, H.  Bryant, A.  Corbett, S.  Steer, J.  Bourke, S. C.  Year: 2016 | Early Supported Discharge/Hospital At Home For Acute Exacerbation of Chronic Obstructive Pulmonary Disease: A Review and Meta-Analysis | Ineligible population:  age profile not specifically older adults and a  Specific disease management (COPD**)** |
| Ghojazadeh, M.  Sanaie, S.  Paknezhad, S. P.  Faghih, S. S.  Soleimanpour, H.  Year: 2019 | Using ketamine and propofol for procedural sedation of adults in the emergency department: A systematic review and meta-analysis | Ineligible population:  Adult not “older adults “ |
| Kwok, E.  Konnyu, K. J.  Skidmore, B.  Moher, D.  Year: 2012 | Effectiveness and safety of emergency department short-stay units: A rapid review | Ineligible population:  Not OA |
| Moe, J.  Kirkland, S. W.  Rawe, E.  Ospina, M. B.  Vandermeer, B.  Campbell, S.  Rowe, B. H  Year: 2017 | Effectiveness of Interventions to Decrease Emergency Department Visits by Adult Frequent Users: A Systematic Review | Ineligible population:  Adult not “older adults “ |
| Pritchard,C.  Ness,A  Symonds, N  Siarkowski,M  Broadfoot,M.  McBrien,KA  Lang, E  Holroyd-Leduc, J  Ronksley, P  Year: 2020 | Effectiveness of hospital avoidance interventions among elderly patients: A Systematic Review | Ineligible population:  Not OA discharged from ED |
| Burkett, Ellen  Martin‐Khan, Melinda G.  Gray, Leonard C.  Year: 2014 | Quality Indicators in the care of the older person in the Emergency Department | Ineligible intervention:  No intervention |
| Carpenter, Christopher R.  Heard, Kennon  Wilber, Scott  Ginde, Adit A.  Stiffler, Kirk  Gerson, Lowell W.  Wenger, Neal S.  Miller, Douglas K.  Year : 2011 | Research priorities for high-quality geriatric emergency care: medication management, screening, and prevention and functional assessment | Ineligible intervention:  Not an intervention |
| Hoon, L. S.  Mackey, S.  Hong-Gu, H.  Year : 2012 | Elderly patients’ experiences of care received in the emergency department: A systematic review | No intervention  Only rated care in the ED. |
| Huntley, A. L.  Chalder, M.  Shaw, A. R. G.  Hollingworth, W.  Metcalfe, C.  Benger, J. R.  Purdy, S.  Year: 2017 | A systematic review to identify and assess the effectiveness of alternatives for people over the age of 65 who are at risk of potentially avoidable hospital admission | Ineligible intervention  Disease Management Specific. The only RCT related to ED intervention is Sun et al 2007 and related to specific management of syncope in ED |
| Iankowitz, N.  Dowden, M.  Palomino, S.  Uzokwe, H.  Worral, P.  Year: 2012 | The effectiveness of computer system tools on potentially inappropriate medications ordered at discharge for adults older than 65 years of age: a systematic review | Ineligible intervention: computer systems management |
| Shapiro, Susan E.  Clevenger, Carolyn K.  Evans, Dian Dowling  Year: 2012 | Enhancing care of older adults in the emergency department | Other: Not an intervention: Commentary on an intervention |
| Vipond, Jennifer  Mennenga, Heidi A.  Year: 2019 | Screening, Brief Intervention, and Referral to Treatment by Emergency Nurses: A Review of the Literature | Ineligible intervention |
| Weigand, J. V.  Gerson, L. W  Year : 2001 | Preventive Care in the Emergency Department: Should Emergency Departments Institute a Falls Prevention Program for Elder Patients? A Systematic Review | Ineligible intervention |
| Chiu, W. K. & Newcomer, R.  Year: 2007 | A systematic review of nurse-assisted case management to improve hospital discharge transition outcomes for the elderly | Ineligible setting (Not Emergency Department) |
| Fan, Lijun  Lukin, William  Zhao, Jingzhou  Sun, Jiandong  Hou, Xiang-Yu  Year: 2015 | Interventions targeting the elderly population to reduce emergency department utilisation | Ineligible setting (not solely focused on OA discharged from ED) |
| Hickman, Louise D.  Phillips, Jane L.  Newton, Phillip J.  Halcomb, Elizabeth J.  Al Abed, Naser  Davidson, Patricia M.  Year: 2015 | Multidisciplinary team interventions to optimise health outcomes for older people in acute care settings: A systematic review | Ineligible setting (Not Emergency Department) |
| Holland, R.  Desborough, J.  Goodyer, L.  Hall, S.  Wright, D.  Loke, Y. K.  Year: 2008 | Does pharmacist-led medication review help to reduce hospital admissions and deaths in older people? A systematic review and meta-analysis | Ineligible setting |
| Oeseburg, B.  Wynia, K.  Middel, B.  Reijneveld, S.  Year: 2009 | Effects of case management for frail older people or those with chronic illness: a systematic review | Ineligible setting (Not Emergency Department)  Setting |
| Stall, N.  Nowaczynski, M.  Sinha, S. K.  Year: 2014 | Systematic review of outcomes from home-based primary care programs for homebound older adults | Ineligible setting (Not Emergency Department)  Setting |
| Weeks, L. E.  Macdonald, M.  Martin-Misener, R.  Helwig, M.  Bishop, A.  Iduye, D. F.  Moody, E.  Year: 2018 | The impact of transitional care programs on health services utilization in community-dwelling older adults: A systematic review | Ineligible setting (Not Emergency Department) |
| Zozula, A.  Carpenter, C. R.  Lipsey, K.  Stark, S.  Year: 2016 | Prehospital emergency services screening and referral to reduce falls in community-dwelling older adults: A systematic review | Ineligible setting (Not Emergency Department) |
| de Vries, M.  Gravel, J.  Horn, D.  McLeod, S.  Varner, C.  Year: 2019 | Comparative efficacy of opioids for older adults presenting to the emergency department with acute pain: Systematic review | Ineligible outcome:  Pain |
| Hesselink, G.  Sir, Ö  Schoon, Y.  Year: 2019 | Effectiveness of interventions to alleviate emergency department crowding by older adults: A systematic review | Ineligible outcome:  ED crowding |
| Riddell, M.  Ospina, M.  Holroyd-Leduc, J. M.  Year: 2016 | Use of femoral nerve blocks to manage hip fracture pain among older adults in the emergency department: A systematic review | Ineligible outcome:  Pain |
| Ritcey, Brandon  Pageau, Paul  Woo, Michael Y.  Perry, Jeffrey J.  Year: 2016 | Regional Nerve Blocks For Hip and Femoral Neck Fractures in the Emergency Department: A Systematic Review | Ineligible outcome:  Pain |
| Steinmiller, J.  Routasalo, P.  Suominen, T.  Year: 2015 | Older People in the Emergency department: a literature review | Factors related to ED visits, not an Intervention |
| Sinha, S  Bessman , E  Flomenbaum, N  Leff, B  Year: 2010 | A Systematic Review and Qualitative Analysis to Inform the Development of a New Emergency Department-Based Geriatric Case Management Model | Ineligible study design |
| Cassarino, M.  Robinson, K.  Quinn, R.  Naddy, B.  O’Regan, A.  Ryan, D.  Boland, F.  Ward, M. E.  McNamara, R.  O’Connor, M.  McCarthy, G.  Galvin, R.  Year: 2019 | Impact of early assessment and intervention by teams involving health and social care professionals in the emergency department: A systematic review | No RCT |
| Jay, S.  Whittaker, P.  McIntosh, J.  Hadden, N.  Year: 2017 | Can consultant geriatrician led comprehensive geriatric assessment in the emergency department reduce hospital admission rates? A systematic review | No RCTs |
| Preston, L.  Chambers, D.  Campbell, F.  Cantrell, A.  Turner, J.  Goyder, E.  Year: 2018 | What evidence is there for the identification and management of frail older people in the emergency department? A systematic mapping review | Ineligible study design: Not a Systematic review |

**References:**

1. Moe J, Kirkland SW, Rawe E, et al. Effectiveness of Interventions to Decrease Emergency Department Visits by Adult Frequent Users: A Systematic Review. *Academic emergency medicine : official journal of the Society for Academic Emergency Medicine* 2017;24(1):40-52. doi: 10.1111/acem.13060 [published Online First: 2016/07/31]

2. Cassarino M, Robinson K, Quinn R, et al. Impact of early assessment and intervention by teams involving health and social care professionals in the emergency department: A systematic review. *PLoS ONE* 2019;14(7) doi: 10.1371/journal.pone.0220709

3. Chiu WK, Newcomer R. A systematic review of nurse-assisted case management to improve hospital discharge transition outcomes for the elderly. *Professional case management* 2007;12(6):330-36; quiz 37-38.

4. de Vries M, Gravel J, Horn D, et al. Comparative efficacy of opioids for older adults presenting to the emergency department with acute pain: Systematic review. *Canadian Family Physician* 2019;65(12):E538-E43.

5. Jay S, Whittaker P, McIntosh J, et al. Can consultant geriatrician led comprehensive geriatric assessment in the emergency department reduce hospital admission rates? A systematic review. *Age and Ageing* 2017;46(3):366-72. doi: 10.1093/ageing/afw231

6. Riddell M, Ospina M, Holroyd-Leduc JM. Use of femoral nerve blocks to manage hip fracture pain among older adults in the emergency department: A systematic review. *Canadian Journal of Emergency Medicine* 2016;18(4):245-52. doi: 10.1017/cem.2015.94

7. Fan L, Lukin W, Zhao J, et al. Interventions targeting the elderly population to reduce emergency department utilisation: a literature review. *Emergency Medicine Journal* 2015;32(9):738. doi: 10.1136/emermed-2014-203770

8. Burkett E, Martin‐Khan MG, Gray LC. Quality indicators in the care of older persons in the emergency department: A systematic review of the literature. *Australasian Journal on Ageing* 2017;36(4):286-98. doi: 10.1111/ajag.12451

9. Carpenter CR, Heard K, Wilber S, et al. Research priorities for high-quality geriatric emergency care: medication management, screening, and prevention and functional assessment. *Academic Emergency Medicine: Official Journal Of The Society For Academic Emergency Medicine* 2011;18(6):644-54. doi: 10.1111/j.1553-2712.2011.01092.x

10. Dickens C, Katon W, Blakemore A, et al. Complex interventions that reduce urgent care use in COPD: a systematic review with meta-regression. *Respiratory medicine* 2014;108(3):426-37. doi: 10.1016/j.rmed.2013.05.011 [published Online First: 2013/06/29]

11. Echevarria C, Brewin K, Horobin H, et al. Early Supported Discharge/Hospital At Home For Acute Exacerbation of Chronic Obstructive Pulmonary Disease: A Review and Meta-Analysis. *COPD: Journal of Chronic Obstructive Pulmonary Disease* 2016;13(4):523-33. doi: 10.3109/15412555.2015.1067885

12. Ghojazadeh M, Sanaie S, Paknezhad SP, et al. Using ketamine and propofol for procedural sedation of adults in the emergency department: A systematic review and meta-analysis. *Advanced Pharmaceutical Bulletin* 2019;9(1):5-11. doi: 10.15171/apb.2019.002

13. Hickman LD, Phillips JL, Newton PJ, et al. Multidisciplinary team interventions to optimise health outcomes for older people in acute care settings: A systematic review. *Archives Of Gerontology And Geriatrics* 2015;61(3):322-29. doi: 10.1016/j.archger.2015.06.021

14. Hoon LS, Mackey S, Hong-Gu H. Elderly patients’ experiences of care received in the emergency department: A systematic review. *JBI Library of Systematic Reviews* 2012;10(23):1363-409.

15. Iankowitz N, Dowden M, Palomino S, et al. The effectiveness of computer system tools on potentially inappropriate medications ordered at discharge for adults older than 65 years of age: a systematic review. *JBI Libr Syst Rev* 2012;10(13):798-831. doi: 10.11124/jbisrir-2012-68 [published Online First: 2012/01/01]

16. Kwok E, Konnyu KJ, Skidmore B, et al. Effectiveness and safety of emergency department short-stay units: A rapid review. *Canadian Journal of Emergency Medicine* 2012;14:S5-S6.

17. Oeseburg B, Wynia K, Middel B, et al. Effects of case management for frail older people or those with chronic illness: a systematic review. *Nursing research* 2009;58(3):201-10.

18. Preston L, Chambers D, Campbell F, et al. What evidence is there for the identification and management of frail older people in the emergency department? A systematic mapping review2018.

19. Purdy S, Huntley A. Predicting and preventing avoidable hospital admissions: A review. *Journal of the Royal College of Physicians of Edinburgh* 2013;43(4):340-44. doi: 10.4997/JRCPE.2013.415

20. Ritcey B, Pageau P, Woo MY, et al. Regional Nerve Blocks For Hip and Femoral Neck Fractures in the Emergency Department: A Systematic Review. *CJEM* 2016;18(1):37-47. doi: <https://dx.doi.org/10.1017/cem.2015.75>

21. Shapiro SE, Clevenger CK, Evans DD. Enhancing care of older adults in the emergency department. *Advanced Emergency Nursing Journal* 2012;34(3):197-203. doi: 10.1097/TME.0b013e31826158bc

22. Sinha SK, Bessman ES, Flomenbaum N, et al. A systematic review and qualitative analysis to inform the development of a new emergency department-based geriatric case management model. *Annals Of Emergency Medicine* 2011;57(6):672-82. doi: 10.1016/j.annemergmed.2011.01.021

23. Stall N, Nowaczynski M, Sinha SK. Systematic Review of Outcomes from Home-Based Primary Care Programs for Homebound Older Adults. *Journal of the American Geriatrics Society* 2014;62(12):2243-51. doi: 10.1111/jgs.13088

24. Šteinmiller J, Routasalo P, Suominen T. Older people in the emergency department: a literature review. *International journal of older people nursing* 2015;10(4):284-305. doi: 10.1111/opn.12090

25. Vipond J, Mennenga HA. Screening, Brief Intervention, and Referral to Treatment by Emergency Nurses: A Review of the Literature. *JEN: Journal of Emergency Nursing* 2019;45(2):178-84. doi: 10.1016/j.jen.2018.10.004

26. Weeks LE, Macdonald M, Martin-Misener R, et al. The impact of transitional care programs on health services utilization in community-dwelling older adults: a systematic review. *JBI Database of Systematic Reviews & Implementation Reports* 2018;16(2):345-84. doi: 10.11124/JBISRIR-2017-003486

27. Weigand JV, Gerson LW. Preventive care in the emergency department: should emergency departments institute a falls prevention program for elder patients? A systematic review. *Academic Emergency Medicine: Official Journal Of The Society For Academic Emergency Medicine* 2001;8(8):823-26.

28. Zozula A, Carpenter CR, Lipsey K, et al. Prehospital emergency services screening and referral to reduce falls in community-dwelling older adults: a systematic review. *Emergency medicine journal : EMJ* 2016;33(5):345-50. doi: 10.1136/emermed-2015-204815 [published Online First: 2016/01/13]

29. Holland R, Desborough J, Goodyer L, et al. Does pharmacist-led medication review help to reduce hospital admissions and deaths in older people? A systematic review and meta-analysis. *British journal of clinical pharmacology* 2008;65(3):303-16. doi: 10.1111/j.1365-2125.2007.03071.x

30. Huntley AL, Chalder M, Shaw ARG, et al. A systematic review to identify and assess the effectiveness of alternatives for people over the age of 65 who are at risk of potentially avoidable hospital admission. *BMJ Open* 2017;7(7) doi: 10.1136/bmjopen-2017-016236

31. Hesselink G, Sir Ö, Schoon Y. Effectiveness of interventions to alleviate emergency department crowding by older adults: A systematic review. *BMC Emergency Medicine* 2019;19(1) doi: 10.1186/s12873-019-0288-4

**Supplementary Information 2 (b)**

**REASONS FOR EXCLUSION: PROTOCOLS REGISTERED ON PROSPERO**

| **CITATION AND REGISTRATION NUMBER** | **TITLE** | **LOG OF EMAIL CONTACT** | **PUBLICATION STATUS** |
| --- | --- | --- | --- |
| Rosalind Elliott, Margaret Fry,  Nicola Wormleaton, Joy Mei  CRD4201811529  2018 | Effective emergency department discharge of older people to home: protocol for a systematic review | 25^th^ May 2020 and 24th September 2020 | Awaiting editorial review in September 2020 |
| Hannah Leaker, Jayna Holroyd-Leduc, Loralee Fox  CRD42018096059  2018 | The impact of geriatric emergency medicine nurses on the care of older patients in the emergency department: a systematic review | 25^th^ May 2020 and 24^th^ September 2020 | Awaiting editorial review in September 2020 |
| Fabrice Mowbray, Komal Aryal,  Mats Junek,  Andrew Costa, Stephanie Sanger  CRD42019125944  2017 | Examining the efficacy of emergency department geriatric case management models: a systematic review and meta-analysis | 25^th^ May 2020 | Discontinued |
| Koen Milisen,  Els Devriendt,  Pieter Heeren, Mieke Deschodt, Marc Sabbe,  Steffen Fieuws, Johan Flamaing, Simon Conroy, Stuart Parker  CRD42015029208  2015 | Effectiveness of comprehensive geriatric assessment based interventions in the  emergency department: a systematic review and meta-analysis | 25^th^ May 2020 | Discontinued |
| Ruth McCullagh,  Joe McVeigh,  Claire Shinkwin  CRD42019122309  2019 | The effectiveness of early supported discharge versus acute hospital care for older adults: a systematic review | 25^th^ May 2020  8^th^ September 2020 | Awaiting Thesis submission September 2020 |
| Merel van Loon,  Britt van Winsen,  M. Christien van der Linden,  Jacobijn Gussekloo, Roos van der Mast  CRD42019141403  2019 | A systematic review of the effectiveness of telephone aftercare for elderly patients after discharge from the emergency department to home | 25^th^ May 2020 and 24^th^ September 2020 | Awaiting editorial review in Emergency Medicine Journal September 2020 |

**Supplementary Information 2 (c)**

**LIST OF EXCLUDED: CONFERENCE ABSTRACTS**

| **CITATION** | **TITLE** | **EMAIL CONTACT** | **STATUS** |
| --- | --- | --- | --- |
| Abbas, M.  D'Sylva, C.  Fraser, L. A. **^1^**  Year: 2016 | Multifactorial assessment and targeted intervention to prevent recurrent falls in community dwelling elderly individuals presenting to emergency departments: A systematic review and meta-analysis. | 25^th^ May 2020  and 1^st^ September 2020 | No response from authors |
| Beales, L. K.^2^  Year: 2016 | “But I'm not a Geriatrician!” incorporating frailty assessment into every encounter in the Emergency Department. | 25^th^ May 2020 | Not published and no usable data |
| Cherian, P.  Rhodes, M.  Mohler, J.  Howe, C.  Sanders, A.  Fain, M. J. ^3^  Year: 2013 | Systematic review of evidence for implementation of geriatric-ED model | 25^th^ May 2020 1^st^ September 2020 | No response from authors |
| Harper, K.  Arendts, G.  Barton, A.  Celenza, A. ^4^  2019 | Fall prevention intervention strategies benefit older people in the emergency department: A systematic review and meta-analysis. | 25^th^ May 2020 and 25^th^ September 2020 | Still under editorial review September |
| Kalim, R.  McMahon, N.  Ryder, S.^5^  Year: 2019 | Pharmacist interventions at discharge and the quality of older patients' care: A systematic review. | 25^th^ May 2020 and 27^th^ September 2020 | Plan to publish but not published as of 27^th^ November 2020. |

**References:**

1. Abbas M, D'Sylva C, Fraser LA. Multifactorial assessment and targeted intervention to prevent recurrent falls in community dwelling elderly individuals presenting to emergency departments: A systematic review and meta-analysis. *Endocrine Reviews* 2016;37(2) doi: 10.1210/endo-meetings.2016.BCHVD.13.SAT-367

2. Beales LK. “But I'm not a Geriatrician!” incorporating frailty assessment into every encounter in the Emergency Department. *European Geriatric Medicine* 2016;7:S96-S97.

3. Cherian P, Rhodes M, Mohler J, et al. Systematic review of evidence for implementation of geriatric-ED model. *Journal of the American Geriatrics Society* 2013;61:S194-S95. doi: 10.1111/jgs.12263

4. Harper K, Arendts G, Barton A, et al. Fall prevention intervention strategies benefit older people in the emergency department: A systematic review and meta-analysis. *EMA - Emergency Medicine Australasia* 2019;31:37. doi: 10.1111/1742-6723.13240

5. Kalim R, McMahon N, Ryder S. Pharmacist interventions at discharge and the quality of older patients' care: A systematic review. *Age and ageing* 2019;48 doi: 10.1093/ageing/afz103.66
