# Supplementary material for: The effectiveness of interventions to reduce adverse outcomes among older adults following Emergency Department discharge: umbrella review
Source: BMC Geriatr. 2022 May 28;22:462. doi: 10.1186/s12877-022-03007-5 (PMC9145107; doi:10.1186/s12877-022-03007-5)
Supplement: Supplementary file 3 — Additional file 3: Supplementary Information 3. Joanna Briggs Institute Critical Appraisal Checklist for Systematic Reviews. [file 12877_2022_3007_MOESM3_ESM.docx]

**Supplementary Information 3: Joanna Briggs Institute Critical Appraisal Checklist for Systematic Reviews**

**JBI Critical Appraisal Checklist for Systematic Reviews and Research Syntheses**

Reviewer Date

Author Year Record Number

|  | Yes | No | Unclear | Not applicable |
| --- | --- | --- | --- | --- |
| 1. Is the review question clearly and explicitly stated? | □ | □ | □ | □ |
| 1. Were the inclusion criteria appropriate for the review question? | □ | □ | □ | □ |
| 1. Was the search strategy appropriate? | □ | □ | □ | □ |
| 1. Were the sources and resources used to search for studies adequate? | □ | □ | □ | □ |
| 1. Were the criteria for appraising studies appropriate? | □ | □ | □ | □ |
| 1. Was critical appraisal conducted by two or more reviewers independently? | □ | □ | □ | □ |
| 1. Were there methods to minimize errors in data extraction? | □ | □ | □ | □ |
| 1. Were the methods used to combine studies appropriate? | □ | □ | □ | □ |
| 1. Was the likelihood of publication bias assessed? | □ | □ | □ | □ |
| 1. Were recommendations for policy and/or practice supported by the reported data? | □ | □ | □ | □ |
| 1. Were the specific directives for new research appropriate? | □ | □ | □ | □ |

Overall appraisal: Include □ Exclude □ Seek further info □

Comments (Including reason for exclusion)
