# Supplementary material for: The effectiveness of interventions to reduce adverse outcomes among older adults following Emergency Department discharge: umbrella review
Source: BMC Geriatr. 2022 May 28;22:462. doi: 10.1186/s12877-022-03007-5 (PMC9145107; doi:10.1186/s12877-022-03007-5)
Supplement: Supplementary file 4 — Additional file 4: Supplementary Information 4. List of Excluded Full text systematic reviews following assessment with Joanna Briggs Institute Critical Appraisal Checklist for Systematic Reviews. [file 12877_2022_3007_MOESM4_ESM.docx]

**Supplementary Information 4:**

**List of Excluded Full text systematic reviews following assessment with Joanna Briggs Institute Critical Appraisal Checklist for Systematic Reviews.**

1. Aminzadeh F, Dalziel WB. Older adults in the emergency department: a systematic review of patterns of use, adverse outcomes, and effectiveness of interventions. *Annals of emergency medicine* 2002;39(3):238-47.
