# Supplementary material for: The effectiveness of interventions to reduce adverse outcomes among older adults following Emergency Department discharge: umbrella review
Source: BMC Geriatr. 2022 May 28;22:462. doi: 10.1186/s12877-022-03007-5 (PMC9145107; doi:10.1186/s12877-022-03007-5)
Supplement: Supplementary file 5 — Additional file 5: Supplementary Information 5. Data Extraction form. [file 12877_2022_3007_MOESM5_ESM.docx]

**Supplementary Information 5:**

**DATA EXTRACTION FORM**

| **CITATION** |  |
| --- | --- |
| **MAIN OBJECTIVE** |  |
| **SEARCH SOURCES, TIME FRAME, LANGUAGE LIMITS** |  |
| **NUMBER OF RCTS INCLUDED IN THE SYSTEMATIC REVIEW** |  |
| **DATE RANGE OF RCTS INCLUDED IN THE SYSTEMATIC REVIEW** |  |
| **COUNTRY OF ORIGIN OF RCTS INCLUDED IN THE SYSTEMATIC REVIEW** |  |
| **PARTICIPANTS** |  |
| **SETTING** |  |
| **INTERVENTION** |  |
| **PROFESSIONAL WHO CARRIED OUT INTERVENTION** |  |
| **CONTROL** |  |
| **CRITICAL APPRAISAL TOOL AND SUMMARY OF ESTIMATE OF THE RCTS INCLUDED** |  |
| **TYPE OF ANALYSES** |  |
| **OUTCOMES AND TOOLS USED TO MEASURE OUTCOMES** |  |
| **NARRATIVE SYNTHESIS** |  |
| **EFFECT SIZE FOR META-ANALYSIS**  **OUTCOMES** |  |
| **FOLLOW UP** |  |
| **COMMENTS** |  |
