# Supplementary material for: The effectiveness of interventions to reduce adverse outcomes among older adults following Emergency Department discharge: umbrella review
Source: BMC Geriatr. 2022 May 28;22:462. doi: 10.1186/s12877-022-03007-5 (PMC9145107; doi:10.1186/s12877-022-03007-5)
Supplement: Supplementary file 6 — Additional file 6: Supplementary Information 6. Table of Characteristics of 9 included systematic reviews. [file 12877_2022_3007_MOESM6_ESM.docx]

**Supplementary Information 6:**

**TABLE OF CHARACTERISTICS OF 9 INCLUDED SYSTEMATIC REVIEWS**

| **CITATION** | **BERNING ET AL 2020** | **CONROY ET AL 2011** |
| --- | --- | --- |
| **MAIN OBJECTIVE** | To summarise interventions that impact the experience of older adults in the emergency department as measured by patient experience instruments. | To examine the evidence for services for older patients who developed a crisis and attended hospital, but who were assessed, treated and discharged, either immediately, or within a short-time period (up to 72 hours) from an *Acute Medical Unit (AMU) or ED |
| **SEARCH SOURCES, TIME FRAME, LANGUAGE LIMITS** | 4 (Ovid Central, Ovid EMBASE, Ovid MEDLINE, PsychINFO), Clinicaltrials.gov.  All searched from Inception to January 16^th^ 2019.  No language restrictions | 11 (OVID Medline , Embase, BNI, HMIC, Cochrane Library, CINAHL, AGEINFO, ASSIA, The National Research Register, National Information Centre on Health Services Research and Health Care Technology (NICHSR), NHS CRD DARE/HTA/EED ).  OVID Medline: 1966- September 2009  EMBASE: 1980- September 2009  BNI: 1985- September 2009  HMIC: Inception to September 2009  Cochrane Library: Inception to September 2009  CINAHL: Inception to September 2009  AGEINFO: Inception to September 2009  ASSIA (Applied Social Sciences Index and Abstracts): inception to September 2009  The National Research Register: Inception to September 2009  National Information Centre on Health Services Research and Health Care Technology (NICHSR): Inception to September 2009  NHS CRD DARE/HTA/EED: Inception to September 2009.  All searched in September 2009 and search rerun in October 2010.  Language restrictions: NR |
| **NUMBER OF RCTS INCLUDED IN THE SYSTEMATIC REVIEW *** | 6 | 5 (4 RCT and 1 Pseudo- RCT) |
| **DATE RANGE OF RCTS INCLUDED IN THE SYSTEMATIC REVIEW** | 1996 to 2013 | 1999 to 2005 |
| **COUNTRY OF ORIGIN OF RCTS INCLUDED IN THE SYSTEMATIC REVIEW** | Australia: 1; Canada: 2; USA: 2, Scotland: 1 | NR |
| **PARTICIPANTS** | N= 1094 (Gagnon et al n= 311, Joubert et al n=8, McCusker et al =229, Mion et a=NR, Runciman et al =414, Wilber et al n = 132). 1 RCT included patients aged ≥ 70 years at risk for repeated hospital admissions. 1 RCT included patients aged ≥ 65 years who screened positive for depression using GDS-15 tool. 1 RCT included patients aged ≥ 65 years with a score of ≥ 2 on the ISAR screening tool. 1 RCT included community dwelling patients aged ≥ 65 years. 1 RCT included patients aged ≥75 years. 1 RCT included patients aged ≥65 years with ability to sit upright. | N= 2287 (n= 1117 Intervention, n= 1170 Control/usual care * error with Table 1 addition, N=2287 IN Table 1, on page number 440 N= 2474).  Age range: ≥ 65 years and ≥ 75 years |
| **SETTING** | ED solely (1 RCT Wilber et al. 2005). 5 RCTS involved pre and post ED care. | Urban ED, home. |
| **INTERVENTION** | Interventions organised via themes: 5 RCTs are described as "Care transitions" evaluating interventions involving care coordination within the ED and care related to post-ED discharge care coordination. 1 RCT described as "Physical needs in the emergency care setting". **Care transitions pre ED discharge care coordination**: Specialised role for conducting pre-discharge assessment and referrals. This was performed by a geriatric social worker, geriatric advanced practice nurse, nurse discharge plan coordinator, or interdisciplinary team (3 RCTs Joubert, Mion and McCusker). **Care transitions (post-ED discharge care coordination):**  Care coordination teams focused on promoting smooth transition following discharge. These teams involved physiotherapy, occupational therapy, speech pathology, nursing, and social work. Home visits were conducted if needed (2 RCT: Gagnon et al , Runciman et al). **Physical needs in emergency care setting:** Reclining hospital chair rather than standard gurney to enhance comfort (Wilber et al 2005)  Specific details on each RCT intervention: 1 RCT Gagnon et al 1996: Coordination of healthcare services by nurse case managers for 10 months following randomisation in the ED. The nurses had at least 2 years of geriatric nursing experience. There were an average of 28 telephone calls and 36 home visits per patient throughout the 10-month intervention.  1 RCT Joubert et al 2013: Community management plan upon discharge, including assessment of outcome reports, patient education about depression by social worker, emotional support and counselling. Contact with general practitioner and health agencies to facilitate post-ED follow-up.  1 RCT McCusker et al 2001: Disclosure of ISAR screening tool results, a brief standardized nursing assessment in the ED, notification of the primary care physician and home care providers, and other referrals as needed.  1 RCT Mion et al 2003: Geriatric assessment by an advanced practice nurse prior to discharge and referral to resources in community (community/social agencies, primary care provider, geriatric clinic) for unmet social and medical needs.  1 RCT Runciman et al 1996: Health visitor screens patients for new dependency and support needs and creates a package of relevant community services. Most patients seen within 24 hours post-discharge from the ED.  1 RCT Wilber et al 2005: Patients were assisted from a standard gurney to a reclining hospital chair by a study nurse. The reclining chair allows patients to sit with their hips and knees flexed at whichever angle they find most comfortable. | CGA  2 RCT: Geriatrician-led CGA focusing on Falls prevention, multifactorial intervention provided on a semi-elective basis in the outpatient department or geriatric day hospital.  3 RCT: Rapid-access, nurse-led, geriatrician-supported comprehensive assessment and management in the community |
| **PROFESSIONAL WHO CARRIED OUT INTERVENTION** | Advanced practice nurse (Mion et al 2003); Research health visitor (Runciman et al 1996); Nurse case manager (Gagnon et al 1999); Nursing assessment plus referral to community home care providers (McCusker et al 2001); Social worker community outreach program (Joubert et al 2013),  Nurse (Wilber et al 2005) | 2 RCTs that were Geriatrician led: 1 RCT included Physiotherapy and Occupational Therapy; 1 RCT included OT home visit. MDT if required.  3 RCTs that were Nurse led: 1 RCT included Nurse and Geriatricians with weekly MDT; 1RCT included geriatrician or emergency physician as required, referrals to community services; 1 RCT included an advanced practice nurse specialising in geriatrics, liaison with emergency staff, and referral to community services. |
| **CONTROL** | Usual Care- * note in Wilber et al 2005 the usual care is a standard gurney | NR |
| **CRITICAL APPRAISAL TOOL AND SUMMARY OF ESTIMATE OF THE RCTS INCLUDED** | Tool used: Modified Cochrane Collaboration Bias Appraisal Tool for RCTs: 3 RCTs judged to be a high risk of bias, 3 RCTs judged as moderate risk of bias. | Tool used: van Tulder critical appraisal tool.  Overall quality of trials: Low. Mean van Tulder score 11.8/19. Range of scores: 10.5-13. |
| **TYPE OF ANALYSES** | Systematic Review with narrative synthesis | Systematic Review with a fixed and a random effects meta-analysis |
| **OUTCOMES AND TOOLS USED TO MEASURE OUTCOMES** | **Patient experience or satisfaction:**  1 RCT (Gagnon et al 1999) utilised Client Satisfaction Questionnaire (CSQ 8): at 10 months post randomisation (Minimum score is 8 and maximum is 32).  1 RCT (Joubert et al 2013) utilised "satisfaction questionnaire" with no details about how it was measured, measured 6 weeks post discharge.  1 RCT (McCusker et al 2001) utilised “Satisfaction with Care Scale” on a 5-point Likert-type scale at 1-month post-enrolment. Possible total scores ranged from 1 to 20 (highest satisfaction).  1 RCT (Mion et al 2003) utilised Satisfaction question on 5-point Likert from poor (1) to excellent (5) at 30-days post-discharge. Number of patients surveyed not described.  1 RCT (Runciman et al 1996) utilised a Satisfaction questionnaire at 4 weeks post-discharge. No details provided on survey questions or measurement.  1 RCT (Wilber et al 2005) utilised Satisfaction questionnaire from 0 (least satisfied) to 10 (most satisfied) scale at two hours following randomisation or upon discharge from the ED. | Mortality: At final follow up (5 RCTs)  Institutionalisation: At final follow up (3RCT)  Functional outcomes: Barthel score (1 RCT)  Quality of Life: SF36 (1 RCT)  Cognition: Mini -Mental State Examination (1 RCT)  Readmissions: Over variable periods of time and at final follow –up (5 RCTS):  Readmission: At 1 month: (3 RCTs) |
| **NARRATIVE SYNTHESIS** | **Patient Experience:**  1 RCT (Joubert et al 2013), showed that the use of a community management plan upon discharge resulted in patients rating health care staff as “wonderful”.  Similarly Mion et al. 2003) showed that geriatric assessment and referral to community prior to discharge by an advanced practice nurse in the ED increased patient satisfaction with the information given (mean score difference 0.37, 95% CI 0.13 to 0.62). McCusker et al. 2001 showed that the disclosure of results of a screening tool to identify seniors at risk did not have statistically significant difference on patient experience outcome (mean 15.12± 3.37 vs. 14.50±3.25). The 2 RCTs (Gagnon et al 1999 and Runciman et al 1996) that focused on interventions involving post ED discharge care coordination did not report improved or higher satisfaction in the intervention groups. Gagnon et al. 1999 did not find any significant difference on patient experience outcome between patients receiving care coordination after ED discharge with a nurse case manager and those receiving usual care (mean difference 1.1, 95% CI−0.1 to 2.3). Runciman et al.1996 described quantitative data that <30% reported improved confidence and self-esteem from the program. One RCT (Wilber et al 2005) reported that patients in the intervention group (offered a reclining chair to sit in) were significantly more satisfied with the reclining chair than the control group were with the standard gurney (mean difference 2.1, 95% CI 1.4 to 2.8). | **Functional outcomes:** MD 0.41, [0.21 to 0.61] favouring intervention via Barthel score (1 RCT).  **Quality of Life: SF36 (1 RCT):** MD 0.2 [**-**1.9 to 2.3] in the physical component of SF36 and favouring intervention and MD of 0.6 [-1.3 to 2.5] in the mental component of SF36. |
| **EFFECT SIZE FOR META-ANALYSIS**  **OUTCOMES** | Narrative synthesis | Mortality: RR 0.92, 95% CI [.55 to 1.52], p=0.767. I² = 0%.  Institutionalisation: RR 0.82, 95% CI [0.53 to 1.28 I² =63.6%.  Readmissions: Full follow up period for all RCTS (5 RCT): RR 0.95, 95% CI [0.83 to 1.08], I² = 42%  Readmission at 1 month: RR 1 95% CI [0.8 to 1.3], I² = 34% |
| **FOLLOW UP** | Range from 2 hours post randomisation to the end of the ED visit up to 10 months post discharge. | Range from 1 month to 18 months |
| **COMMENTS** | There was significant heterogeneity in the tools used to measure patient experience. Overall, low certainty in the evidence available highlights the need for more reliable tools to measure patient experience and studies designed to measure the effect of the interventions. | There were limited number of trials evaluating the role of CGA in frail older adults being discharged rapidly from ED. High heterogeneity requires caution when interpreting results. |
| **CITATION** | **FEALY ET AL 2009** | **HASTINGS & HEFLIN 2005** |
| **MAIN OBJECTIVE** | 1. How effective are gerontologically informed nursing assessment and referral interventions aimed at older Emergency Department attendees?  2. What are the methodological issues associated with evaluating the effectiveness of these interventions? | To evaluate the evidence for interventions designed to improve outcomes for elders discharged from the Emergency Department. |
| **SEARCH SOURCES, TIME FRAME, LANGUAGE LIMITS** | 4 (CINAHL, MEDLINE (PubMed), Science Direct, Cochrane Central Register of Controlled Trials (CENTRAL))  CINAHL: 1992-31 August 2008  MEDLINE (PubMed): 1992-31 August 2008  Science Direct: 1992-31 August 2008  Cochrane Central Register of Controlled Trials (CENTRAL): 1992-31 August 2008  Search done: 31st August 2008  Language restrictions: English only | 2 (MEDLINE, CINAHL)  MEDLINE: 1966 to January 2005  CINAHL: 1966-January 2005  Search done: January 2005  Language restrictions: English only |
| **NUMBER OF RCTS INCLUDED IN THE SYSTEMATIC REVIEW** | 6 (5 RCT and 1 Quasi Pseudo- RCT) | 6 (5 RCT and 1 Pseudo- RCT) |
| **DATE RANGE OF RCTS INCLUDED IN THE SYSTEMATIC REVIEW** | 1996 to 2005 | 1999 to 2004 |
| **COUNTRY OF ORIGIN OF RCTS INCLUDED IN THE SYSTEMATIC REVIEW** | USA: 1 RCT  Australia: 2 RCT  Scotland: 1 RCT  Canada: 2 (1 RCT and 1 Quasi RCT) | Canada: 3 (2 RCT and 1 Pseudo RCT)  USA: 1 RCT  Australia: 1 RCT  Scotland: 1 RCT |
| **PARTICIPANTS** | N= 2852  ≥ 65 years (2 RCT)  ≥70 years (1RCT)  ≥ 75 years (2 RCT)  “Elderly” (1 RCT)  No specific age range given (1RCT) | N given for Caplan et al. 2004 only (N= 739)  2 RCT: Community dwelling ED patients aged 75 years and over discharged home.  1 RCT: Community-dwelling ED patients aged 65 years expected to be discharged home.  1 quasi RCT: Community-dwelling ED patients aged 65 years expected to be discharged home; designated as high risk by ISAR screening instrument.  1 RCT: Community-dwelling ED patients aged 70 years at increased risk for hospital admission (Boult assessment  tool) with functional dependence; discharged home.  1 RCT: Adults being discharged from the ED with a change in care requirements (63% aged 65 years). |
| **SETTING** | Urban ED | 2 RCTs: Urban ED  1 RCT: Single centre in Scotland  1 RCT: Community hospital ED  1 RCT: University hospital affiliated ED  1 RCT: University Hospital |
| **INTERVENTION** | 1 RCT: ED based CGA by an aged care specialist nurse, carer and healthcare provider liaison and post discharge referrals.  1 RCT: Nursing assessment at an ED and at home incorporating care planning, urgent interventions and referrals, weekly interdisciplinary case presentation, initiation of appropriate interventions for up to 4 weeks.  1 RCT: Comprehensive case finding assessment and referral intervention conducted by an advanced practice nurse during an ED visit.  1 RCT: ISAR screening tool administered by community nurse either by interview in the ED or by telephone post discharge.  1 RCT: Nurse case management. Minimum of a monthly telephone call and home visit every 6 weeks.  1 RCT: Visit by a health visitor who conducted a standardised assessment and screening for new dependency and support needs, and referred to appropriate community services. | 1 RCT: CGA performed during nurse home visit and follow-up to four weeks; review with MDT  1 RCT: CGA in the ED; discharge plan with referrals, summary to primary care provider, short-term follow-up with home care agencies.  1 RCT: StandardiSed geriatric nursing assessment, referrals, routine notification of primary care provider, limited follow-up.  1 RCT: Nurse case management; minimum of a monthly phone call and home visit every six weeks.  1 RCT: Home care coordinators in the ED arranged prompt delivery of multidisciplinary in-home services.  1 RCT: Home visit by health visitor for standardized assessment and referral to community service. |
| **PROFESSIONAL WHO CARRIED OUT INTERVENTION** | All trials relate to Nurses | 2 RCTs: Nurse carried out the CGA  1 RCT: Home care coordinators  1 RCT: Health visitor |
| **CONTROL** | Usual Care | Usual Care |
| **CRITICAL APPRAISAL TOOL AND SUMMARY OF ESTIMATE OF THE RCTS INCLUDED** | Tool used: Checklist described by Grimshaw et al 2003.  No fundamental methodological flaws in study designs 4 RCTs: of comparable quality. | An assessment of methodological validity of the clinical trials was performed - no specific tool reported.  4 RCTs: of comparable quality |
| **TYPE OF ANALYSES** | Systematic Review with narrative synthesis | Systematic Review and narrative synthesis |
| **OUTCOMES AND TOOLS USED TO MEASURE OUTCOMES** | Admission to hospital (4 RCTS):  Length of stay in hospital (2 RCTS): days  Nursing home placement/admission (2 RCTS):  Functional decline (5 RCTS): Variety of measurement tools including dependence with ADL and IADL  Quality of Life: SF36 (1 RCT)  Patient and care giver satisfaction (1 RCT)  Mortality  Readmissions: Full follow up period for all RCTS (5 RCT)  Readmission to ED (3 RCTS) | Functional decline: 1 RCT used IADL and BADL ADL, IADL dependence; 2 RCT used OARS, 1 RCTS used Barthel Index,  ED readmission: 30 days to 180 days  Hospital admission: 30 DAYS  Institutionalisation: At follow up.  Death: At follow up.  Patient experience: 1 RCT utilised Client satisfaction questionnaire. No tool mentioned for 2 RCTs. |
| **NARRATIVE SYNTHESIS** | 1 RCT (Caplan et al. (2004)) reported statistically significantly fewer elective and emergency admissions for intervention participants [61 vs. 82 P = 0.048], and a statistically significant decrease in the number of emergency admissions for intervention participants [164 vs. 201 ; P = 0.0072] over the 18 months of their follow-up intervention.  1 RCT (Mion et al. (2003)) demonstrated effectiveness in lowering service use: at 30 days after the index ED visit, intervention participants were less likely than controls to have nursing home admission (OR 0.21; 95% CI: 0.05 to 0.99). High-risk intervention participants had fewer hospital days (mean difference of -1.0, 95% CI: -2.0 to 0.0), fewer nursing home admissions (OR 0.2 , 95% CI: 0.04 to 0.96) at 30 days, and continued to have lower nursing home admissions (OR 0.3 95% CI: 0.07 to 0.94) at 120 days when compared with usual care high-risk participants. There was a statistically significant greater number of community agency referrals observed among intervention participants. A similar observation was reported by McCusker et al 2003A in that intervention participants were more likely to have a referral to a local health centre and to receive home care services at one month post ED visit. However, intervention participants were also more likely to make a return visit to the ED (OR 1.6, 1.0 to 2.6).  1 RCT (Caplan et al. (2004) reported less decline in physical function among intervention participants at 6 months in Barthel scores. Physical function was similar for both groups at 18 months. McCusker et al.  (2001) observed a statistically significantly reduced rate of functional decline at 4 months among intervention participants (adjusted OR 0.60, [0.36 to 0.99).  1 RCT (Runciman et al 1996): intervention participants were statistically significantly more independent in instrumental ADL at 4 weeks post discharge. | 3 RCTS reported a reduction in functional decline at different time points, (range from 4 months to 18 months) and using different measurement tools (dependence with ADLs and IADLs, Barthel). 1 RCT reported no significant difference in functional status and another reported no significant difference. The value of screening for high-risk patients may be more effective than applying intense interventions more broadly. There were mixed results in service utilisation with one RCT showing reduction in hospital admission at 30 days and emergency hospital admission at 18 months. 2 RCTs showed an increase in unscheduled ED visits (Note-McCusker et al 2001 used 30 days, Gagnon et al 1999 measured at 10 months).  3 RCTs reported no significant change in QOL or mortality. 4 RCTS reported no statistical difference in patient experiences. |
| **EFFECT SIZE FOR META-ANALYSIS**  **OUTCOMES** | Narrative synthesis | Narrative synthesis |
| **FOLLOW UP** | 4 weeks to 18 months | 1 month to 18 months |
| **COMMENTS** | CGA and post discharge referral may reduce service use, including hospital admissions, re-presentation to the ED but results need to be interpreted with caution.  A number of trials have failed to demonstrate effectiveness in predicted patient and/or healthcare systems outcomes and in some instances have identified increased service use (nursing assessment and referral interventions) and may lead to reduced functional decline. | Functional decline in high risk elders can be reduced using geriatric nursing assessment and home based services.  Results of trials to decrease health service utilisation rates following an ED visit were mixed. |

| **CITATION** | **HUGHES ET AL 2019** | **KARAM ET AL 2015** |
| --- | --- | --- |
| **MAIN OBJECTIVE** | How effective are ED interventions in improving clinical, patient experience, and utilisation outcomes in Older adults 65 years of age and older? | To review the literature on ED-based interventions and examine the evidence on reductions in ED re-visits, hospitalizations, nursing home admissions and deaths among older adults.  To determine if previously identified gaps in the research have since been addressed, and make recommendations to improve intervention design and evaluation methods based on a framework of intervention classifications developed in the present review. |
| **SEARCH SOURCES, TIME FRAME, LANGUAGE LIMITS** | 4 (Medline, Embase, CINAHL, PsychINFO).  Search range not reported  Search done: December 2017  Language restrictions: English only | 4 (Medline (PubMed), CINAHL, Embase and Web of Science).  Search range not reported  Search done: June 2012  Language restrictions: English only |
| **NUMBER OF RCTS INCLUDED IN THE SYSTEMATIC REVIEW** | 8 RCTs, 1 cluster-randomised study | 3 |
| **DATE RANGE OF RCTS INCLUDED IN THE SYSTEMATIC REVIEW** | 1996 to 2017 | 2003 to 2007 |
| **COUNTRY OF ORIGIN OF RCTS INCLUDED IN THE SYSTEMATIC REVIEW** | Australia: 2  Europe: 2  USA :3  Canada: 2 | Australia: 1  Canada: 1  USA: 1 |
| **PARTICIPANTS** | N= 4561 randomised (both high risk and unselected older adults) 4 studies high risk patients only, 5 studies unselected patients.  59% female  Median patient age (range): 79 (74-82) (1 study NR).  Patients with cognitive impairment: 27.3% (% studies NR) | N= 1475  1 RCT: N= 739, Intervention n = 370, Control N= 369. Age ≥ 75 years, discharged from ED.  1 RCT: N= 650, Intervention n = 326, Control N= 324. Age > 65 years, discharged from ED, lived within the community, had telephone access, had a family member willing to act as proxy in cases of severe cognitive impairment. Triage Risk Screening tool used to stratify patients into risk groups (High and Low).  1 RCT: N= 86, Intervention N= 43, Control N=43. Age ≥ 70 years, Presented to ED after a fall, lived in their own house before fall, had telephone service, mentally competent, lived within geographic catchment area. |
| **SETTING** | Urban ED | No details regarding study setting except intervention in ED and home |
| **INTERVENTION** | 4 *single* strategy interventions (Case Management/Transition of Care).  5 *multi*-strategy interventions (3 Discharge planning PLUS Case Management/Transition of Care 2 Case Management/Transition of Care PLUS Medication Management.)  **Discharge planning** is time-limited, taking place fully within the ED, and encompassing the process of thinking about and formaliSing a plan of care prior to a patient’s discharge from the ED. Discharge planning may incorporate 1 or more of the following: geriatric consultation or geriatric assessment in the ED, patient/caregiver education, or a follow-up plan. Although the initial assessment and discharge planning take place within the ED, the responsibility for coordinating and obtaining follow-up care rests with the patient or caregiver.  **Case management** takes place over time and across settings, initially beginning within the ED and continuing after discharge, and includes the activities that a physician or other health care professional performs to ensure coordination of medical services needed by the patient. The ultimate goal of case management is to help support successful transition from the ED to post-ED settings. Unlike discharge planning in which the patient or caregiver may be responsible for identifying and securing services, in case management, the major responsibility and coordination rests with 1 or more providers.  Interventions that assist patients or caregivers in managing and monitoring drug therapy for older adults with chronic conditions.  **Medication safety or Management:** Interventions that assist patients or caregivers in managing and monitoring drug therapy for older adults with chronic conditions.  **Multi-strategy interventions**: Two or more intervention strategies (e.g., discharge planning and case management, discharge planning and medication safety) | 2 RCT: CGA in the ED plus referrals to various community resources. One of the RCT also incorporated TRST to stratify patients into Low risk and high risk.  1 RCT involved Personal Emergency Response System (PERS). |
| **PROFESSIONAL WHO CARRIED OUT INTERVENTION** | 4 Single Providers: 4 Nurse  5 Multiple different Providers: 1 MD and Nurse and PT; 1 PT; 1 RN; 1 MD and RN and SW; 1 RN and SW | 2 RCT: Nurse (Advanced practice Nurse), referral to various community resources. 1 RCT: NR |
| **CONTROL** | Usual Care | Usual Care |
| **CRITICAL APPRAISAL TOOL AND SUMMARY OF ESTIMATE OF THE RCTS INCLUDED** | Cochrane EPOC ROB  Objective outcomes: 2 high risk, 2 unclear risk, 2 low risk, 1 N/A  Subjective outcomes: 4 high risk, 3 unclear risk, 2 low risk. | NR |
| **TYPE OF ANALYSES** | Systematic Review with random effects meta analysis and narrative synthesis | Systematic Review and narrative synthesis |
| **OUTCOMES AND TOOLS USED TO MEASURE OUTCOMES** | ED readmission: 7 RCT  Functional status : 6 RCTs  Hospital admission: 5 RCT  Patient experience: 4 RCT  QOL: 2 RCT | ED readmission/revisits: 3 RCTs inform this outcome (N= 1475)  Hospital admission: 3 RCTs inform this outcome (N= 1475)  Mortality: 2 RCTs inform this outcome ( N= 1389)  Nursing Home admission: 1 RCT informs this outcome (N= 650) |
| **NARRATIVE SYNTHESIS** | Functional Decline: Positive intervention effects were observed in four of the five RCTs Functional status was assessed differently across the studies. Three RCTS defined functional status as changes in dependence in activities of daily living (ADLs) or instrumental activities of daily living (IADLs). One multi-strategy study, using discharge planning plus case management  and one key intervention component, found a statistically significant lower odds of clinically important functional  dependency (odds ratio [OR] = .53; 95% CI = .31-.91) at 3 and 4 months, respectively. A second multi-strategy study, also utilizing discharge planning plus case management and one key intervention component, found a significantly greater odds of functional improvement, as defined by improvement  in ADL performance measured at 3 months (OR = 2.37; 95% CI = 1.20-4.68) and 12 months (OR = 2.04; 95% CI =  1.03-4.06). A third single-strategy study of case management involving one key intervention component found that intervention participants reported higher levels of functional independence  in IADLs compared with the control group (P = .027), but there were no significant differences in ADL independence  (P = .47).  Quality of Life:  Two RCTS evaluated multi-strategy interventions of discharge planning plus case management on QOL. Both studies reported physical and mental  health–related QOL using the Short Form-36 physical function and mental health component scores at 30 and  120 days. There were no statistically significant effects of the ED interventions on either physical or mental health– related QOL at any time point.  Patient experience: 4 RCTs reported mixed effects using a range of outcome measures. Two studies evaluated multi-strategy interventions of discharge planning plus case  management, and they included all three intervention components. Overall, these studies show a mixed pattern, with one single-strategy and one multi-strategy  RCT reporting higher satisfaction with care or greater patient knowledge of community resources. Two RCTS, one using case management and the second  discharge planning plus case management, evaluated patient satisfaction with care using continuous outcome measures, the Client Satisfaction Questionnaire and Satisfaction with Care Scale. Assessment time points occurred at 1 and 10 months. There were no statistically significant effects on  patient experience in either study. A third RCT found higher satisfaction among intervention participants regarding  Information received in the ED on postdischarge support services. A fourth RCT  evaluating case management utilized an unnamed instrument and found that 40% of intervention participants recalled  helpful information, and 28% reported benefits of improved confidence and self-esteem. | Regarding CGA, reductions in hospitalizations were found at follow up (30 days: 11.9% intervention vs 14.4% control, difference in percentage −2.5, 95% CI −7.4 to 2.4, P = 0.312; 18 months: 44.4% intervention vs 54.3% control, DP −9.9, 95% CI −17.1 to −2.7, P = 0.007) were reported in 1 RCT while there was no difference in this outcome in the other RCT utilising CGA. Lower rates of ED revisits were also reported in 1 RCT but not observed in the other. The RCT which stratified patients into risk groups reported that the intervention group had fewer admissions to nursing homes when compared with the randomly selected control group at 30- and 120-day follow up (30 days: 2% intervention vs 7% control, odds ratio [OR] 0.2, 95% CI 0.04–0.96; 120 days: 3% intervention vs 10% control, OR 0.3, 95% CI 0.07–0.94). Within the low-risk cohort, there was also found an overall reduction in nursing home admissions (30 days: 0.7% intervention vs 3.0% control, OR 0.21, 95% CI 0.05–0.99). No differences were observed in mortality.  The RCT which utilised PERS Device reported no difference between subsequent ED re-visits (day 60–67: 19% intervention vs 19% control, risk difference 0.0%, 95% CI −16 to 16%, P = 1.0) and only a slight change in hospitalisations (day 60–67: 7% intervention vs 14% control, risk difference −7.0%, 95% CI −19.8% to 5.9%, P = 0.29). |
| **EFFECT SIZE FOR META-ANALYSIS**  **OUTCOMES** | Hospital admission after the index visit: RR 0.96 [0.51 to 1.83] I² = 63.25% -Dicot outcome, there was no intervention effect for the randomised studies.  Three RCTS reported hospitalisation after the ED index visit using a variety of continuous outcome measures. Only one study, which used all three intervention components, found a significant effect of the intervention  on hospitalisation after the ED index, with a  reported number needed to treat of 18 to prevent one hospitalization at 30 days, and a number needed to treat of 10 to prevent one hospital admission at 18 months.  Emergency department return visit: Relative Risk (RR) 1.13,[.94 to 1.36] P= 0.41. : I² = 0.9%, | Narrative synthesis |
| **FOLLOW UP** | 18 months | 3, 6, 12, 18 months |
| **COMMENTS** | There was a mixed effects of ED interventions on select clinical and utilisation outcomes.  Limited number of studies evaluating single intervention strategy makes it difficult to make definitive conclusions regarding their effectiveness. Individual studies investigating multi strategy intervention (3 or more components) may be associated with small benefits in functional status, decreased hospitalisation after the ED index visit, and a lower likelihood of ED return visits. | There was substantial variation in the study methods. Interventions that incorporated a geriatric assessment within the ED and follow up by a geriatric care team showed some success, but were not consistent across all outcomes. |

| **CITATION** | **LOWTHIAN ET AL 2015** | **MALIK ET AL 2018** |
| --- | --- | --- |
| **MAIN OBJECTIVE** | (1) Profile effective ED-based care transition models  2) Provide robust estimates of effect of these care models on risk of ED re-presentation or hospitalisation, functional decline in ADL, nursing-care home admission and mortality in older people discharged home from the ED | To appraise the impact of geriatric focused nurse assessment and interventions in the ED in terms of admission rate, ED revisits and length of hospital stay. |
| **SEARCH SOURCES, TIME FRAME, LANGUAGE LIMITS** | 2 ( OVID Medline, Cochrane Library)  OVID MEDLINE: 1946-2013  Cochrane Library: 2005-2013  Clinicaltrials.gov  Search done: December 2013.  No language restrictions | 6 ( Cochrane, Medline, CINAHL, EMBASE, Scopus, Web of Knowledge)  All databases: 1990 to 2016  Search done: The last date searched was May 2016  Language restrictions: English only |
| **NUMBER OF RCTS INCLUDED IN THE SYSTEMATIC REVIEW** | 5 RCTS (4 RCT, 1 Quasi RCT)  *1 RCT (Yim et al. 2001 did not inform outcomes)* | 7 |
| **DATE RANGE OF RCTS INCLUDED IN THE SYSTEMATIC REVIEW** | 1996 to 2011 | 1996 to 2015 |
| **COUNTRY OF ORIGIN OF RCTS INCLUDED IN THE SYSTEMATIC REVIEW** | Australia: 1  Canada: 1  USA: 1  Scotland: 1  Hong Kong: 1 | Australia: 2  Canada: 2  USA: 1  Scotland: 1  Denmark: 1 |
| **PARTICIPANTS** | N= 3447 randomised (both high risk and unselected older adults).  2 RCTS included high-risk patients (n= 1279 total).  Age range: ≥65 to ≥75 | Study participants were of both sexes ≥65 years who underwent a geriatric focused nurse assessment/intervention in EDs or following discharge.  Numbers for each RCT are not given |
| **SETTING** | 5 RCTS: ED and community | Patients were recruited from EDs of university based hospitals in 4 RCTS, general hospitals in 1 RCT, a tertiary cardiac hospital in 1 RCT and medical school affiliated public hospital in an urban setting in 1 RCT. 4 RCTs were performed in the ED prior to discharge with follow-up assessment at home ; 1 RCT was performed in ED for both admitted and discharged patients with no follow-up assessment in the community. In 2 RCTS a post ED discharge a comprehensive assessment was conducted at home. |
| **INTERVENTION** | 1 RCT: Comprehensive geriatric nurse  Assessment + home visit within 24 hours, plus formulation of discharge care plan, plus referrals, plus GP liaison, plus 4-week home-based intervention, + weekly interdisciplinary team meetings, + seamless transfer of care to  community-based services.  1 RCT: Health visitor home visit and assessment within 24 hours of ED discharge with development of community services package + discussion with patient and GP + service provision arrangement.  1 RCT: Comprehensive geriatric nurse assessment plus formulation of discharge care plan, plus referrals, plus GP liaison, plus telephone follow-up until community-based services transfer.  1 RCT: ISAR screening to identify high-risk  patients + brief standardised geriatric  nursing assessment, + team discharge care planning + community services referrals. | "Geriatric focused nurse assessment and intervention". A range of assessments and interventions were employed in the 7 RCTs. These included nurse assessment using a disease management risk scale and/or various risk screening tools in ED with follow up care in the community post discharge in RCTs. In 2 RCTS patients received a CGA by an Advanced Nurse Practitioner specialising in geriatrics and a multidisciplinary approach to identify unresolved medical, healthcare or social needs. 1 RCT described Nurse case management, which consisted of coordination and provision of healthcare services by nurses, both in and out of hospital, for a 10‐month period. One RCT evaluated impact of health visitor follow-up for elderly patients discharged from ED assessing services provided, patient satisfaction, readmission rates, dependency and functional outcomes. |
| **PROFESSIONAL WHO CARRIED OUT INTERVENTION** | 3 RCTS: Nurse carried out geriatric assessment  1 RCT: Health visitor home visit | Interventions were focused on a geriatric focused nurse assessment/intervention. No specific mention of other professionals "provision of health care services". 2 RCTs refer to multidisciplinary approach" |
| **CONTROL** | Usual Care | Usual Care |
| **CRITICAL APPRAISAL TOOL AND SUMMARY OF ESTIMATE OF THE RCTS INCLUDED** | Tool used: Cochrane Collaboration Risk of Bias tool.  2 RCTS : Low ROB  1 RCT : High ROB  1 RCT : Moderate ROB | Risk of Bias tool on Revman 5.2 risk of bias tool. There is a lack of clarity regarding allocation concealment in four RCTs. Inadequate blinding was another methodology concern in some studies. In Basic et al 2015, the age care nurse involved in early assessment was aware of group allocation as were the patients involved. Furthermore, the age care nurse measured all outcome data similar to Caplan et al. Where person assessing the participant measured outcome data. Some RCT acknowledged blinding of subjects and assessors was unfeasible given the nature of the intervention with other trials not stating if blinding took place signifying an unclear risk of bias. All RCTs had a high risk of attrition bias. |
| **TYPE OF ANALYSES** | Systematic Review and Random effects Meta-analysis | Systematic Review and meta-analysis |
| **OUTCOMES AND TOOLS USED TO MEASURE OUTCOMES** | Functional decline: in ADL 4 weeks to 18 months. Measured in 2 RCTs no pooling of results due to diversity of measures and methods of outcome reporting.  Tools used: Barthel, IADL.  ED revisit: 1 month post ED discharge  Emergency hospital admission 2 RCTS: 1 month after initial attendance  Mortality: 2 RCTs at 18 months post ED discharge | Hospital readmission 3 RCTs informed this outcome: 30 days to 180 days post ED visit  ED revisits 3 RCTs informed this outcome |
| **NARRATIVE SYNTHESIS** | Functional Decline: Measured in 2 RCTS using different tools and at at different time points (6 and 12 months in Caplan et al 2004 and 1 and 4 months in Mion et al 2003. Caplan et al 2004 compared changes in functional status as a secondary outcome, reporting a greater degree of independence in ADL at 6 months and no decline in cognitive function at 12 months in the ED-CTS group. At 18 months, deterioration in ADL and cognitive status was reported for both groups. Mion reported no impact on physical or mental function (SF 36)  with the intervention at either time point. Tools used: Barthel, IADL in Caplan et al 2004. |  |
| **EFFECT SIZE FOR META-ANALYSIS**  **OUTCOMES** | Unplanned ED re-presentation: 1 month: OR 1.32. 0.99-1.76, p 0.06  I²= 0%  Emergency hospital admission: I²= 0%  Mortality: OR 1.01. 0.70-1.47, p= 0.94  I²= 0%  NH admission | Hospital admissions: Pooled data of 3 RCTS that measured outcome at day 30 post-intervention did favour the Intervention. OR: 0.84, 95% CI: 0.70 to 1.02,  *I*² =35%.  ED revisit: OR: 1.03, 95%CI: 0.84 to 1.26. I²= 62% |
| **FOLLOW UP** | One month to 18 months | 30 days to 10 months |
| **COMMENTS** | Limited high quality data to guide recommendations. There is limited evidence for effectiveness in reducing unplanned ED attendance, hospital admission or mortality | Nursing focused geriatric assessment and interventions did not have a significant statistical impact on rates of hospitalisation, readmission, ED revisits. Numerous methodological issues should be considered when reviewing these results. There is a lack of clarity regarding allocation  concealment in four RCTs -indicates selection bias. Heterogeneity of RCTs with different types of assessment tools used and varied interventions utilised. |

| **CITATION** | **MORELLO ET AL 2019** |
| --- | --- |
| **MAIN OBJECTIVE** | To determine the effects of multifactorial falls prevention interventions on falls, fall injuries, fractures, Emergency Department presentations and hospitalisations in older adults presenting to the Emergency Department with a fall. |
| **SEARCH SOURCES, TIME FRAME, LANGUAGE LIMITS** | 5 ( OVID Medline, CINAHL, EMBASE, PEDro, Cochrane Central Register of Randomised Controlled Trials).  All databases searched from Inception to June 2018.  Search done: June 2018  No language restrictions |
| **NUMBER OF RCTS INCLUDED IN THE SYSTEMATIC REVIEW** | 12 |
| **DATE RANGE OF RCTS INCLUDED IN THE SYSTEMATIC REVIEW** | 1999 to 2018 |
| **COUNTRY OF ORIGIN OF RCTS INCLUDED IN THE SYSTEMATIC REVIEW** | Australia: 4  UK: 4  Netherlands: 1  Singapore: 1  Hong Kong: 1  Denmark: 1 |
| **PARTICIPANTS** | N= 3986. The median number of participants randomised per trial was 340 (range 109 to 712). Mean age was 78 years and ranged across studies from 73 to 84 years. Average gender mix was 69% women (ranging from 55% to 80%). |
| **SETTING** | Assessments were undertaken in a variety of settings and on occasion by more than one health professional, including the participant’s home (10 studies), an outpatient setting for example, day hospital or clinic (2 RCTs) No detail reported regarding the setting as urban or rural. |
| **INTERVENTION** | All RCTs included an assessment of falls risk factors. Assessment tools and risk factors assessed varied considerably across studies. The most common falls risk factors assessed were home environment (10 studies), mobility or gait (nine studies), vision (10 studies) and balance (seven studies). Assessments were undertaken in a variety of settings and on occasion by more than one health professional, including the participant’s home (10 studies), an outpatient setting for example, day hospital or clinic (four studies) or as an inpatient (two studies). The specific interventions delivered were highly variable, including education (11 studies), referral to healthcare services (11 studies), home modifications (eight studies), exercise (six studies) and medication change (five studies). Some studies provided only limited treatment options, such as education and referral to healthcare services, whereas others provided many potential intervention strategies. The time until delivery of intervention was reported in only six studies, and ranged from 2 to 8 weeks after completion of baseline assessment. Frequency of the recommended interventions varied from 1 to up to 16 sessions. |
| **PROFESSIONAL WHO CARRIED OUT INTERVENTION** | Interventions were led by a variety of healthcare professionals, including occupational therapists, physiotherapists,  registered nurses and medical professionals. |
| **CONTROL** | Usual Care |
| **CRITICAL APPRAISAL TOOL AND SUMMARY OF ESTIMATE OF THE RCTS INCLUDED** | The PEDro scale.  RCTs were of variable methodological quality. Methodological strengths included allocation concealment and between- group comparisons for analysis. Common limitations were the lack of blinding and inadequate follow-up of participants. Range in PEDro scale 5- 8 (out of a possible 11). |
| **TYPE OF ANALYSES** | Systematic Review and Random effects Meta-analysis |
| **OUTCOMES AND TOOLS USED TO MEASURE OUTCOMES** | Rate of falls: Falls calendars or diaries (9 RCTS informed this outcome)  Number of fallers: Falls calendars or diaries (12 RCTS informed this outcome)  Neck of femur fractures: Medical records (3 RCTS informed this outcome)  Falls related ED presentations: Medical records (3 RCTS informed this outcome).  Falls related hospitalisation: Medical records (3 RCT informed this outcome  Falls injuries and injurious falls (8 RCTs informed this outcome  Other fractures (2 RCTs informed this outcome): |
| **NARRATIVE SYNTHESIS** | N/A |
| **EFFECT SIZE FOR META-ANALYSIS**  **OUTCOMES** | Rate of falls: Falls calendars or diaries (9 RCTs informed this outcome): RR 0.78, 0.58 to 1.05, p value=0.1, I² = 94%.  Subgroup analyses demonstrated that studies that included two or more interactions in their multifactorial intervention programme had a significant reduction in the rate of falls (rate ratio: 0.62, 95% CI: 0.45 to 0.86). RCTs which included treatment of risk factors, rather than just referral-based interventions, demonstrated a significant  reduction in the rate of falls (rate ratio: 0.78, 95% CI: 0.58 to 0.93). No difference was observed for number of fallers in these subgroups.  Number of fallers: Falls calendars or diaries (12 RCTS informed this outcome): RR 1.02, 0.88 to 1.18, p 0.77, *I*² = 75%.  Neck of femur fractures: Medical records (3 RCTS informed this outcome): RR 0.82, 0.53 to 1.25, p 0.35. *I²* = 0%.  Falls related ED presentations: Medical records (3 RCTS informed this outcome):  RR 0.99, 0.84 to 1.16, p 0.86*, I²=* 33%.  Falls related hospitalisation: Medical records (3 RCT informed this outcome): RR 1.14, 0.69 to 1.89, 0.6, *I²*= 58%.  Falls injuries and injurious falls (8 RCTs informed this outcome): 8 RCTs that reported on either injurious falls or fall injuries, no study observed a statistically significant effect.  Other fractures (2 RCTS informed this outcome): 1 RCT reported a reduction in the rate of fractures in the intervention group (incidence rate ratio: 0.37, 95% CI: 0.15 to 0.91). The other RCT observed no effect on fractures. |
| **FOLLOW UP** | 6 to 12 months |
| **COMMENTS** | Multifactorial intervention programmes did not reduce falls in older people who present to the ED with a fall, unless the programme included two or more interactions, or the treatment of risk factors, rather than referral-based interventions alone. No significant reductions were observed in the number  of fallers, fractured neck of femurs, ED presentations or hospitalisations with the delivery of multifactorial falls prevention  programmes. Heterogeneity of multifactorial interventions, inconsistent definition of outcomes between RCTs. |

**Abbreviations:**

ADL: Activities of Daily Living

AMU: Acute Medical Unit

BADL: Basic Activities of Daily Living

CGA: Comprehensive Geriatric Assessment

CI: Confidence Intervals

ED: Emergency Department

EPOC: Effective Practice and Organisation of Care

*et al.:* et alii, meaning “and others”

GP: General Practitioner

ISAR: Identification of Seniors at Risk

MD: Medical Doctor

MDT: Multidisciplinary Team

N/A: Not applicable

NR: Not reported

OARS: Older Americans Resources and Services

OR: Odds ratio [OR]

OT: Occupational Therapist

PERS: Personal Emergency Response System (PERS

PT: Physical Therapist

QOL: Quality of Life

RCT: Randomised controlled trial

RN: Registered Nurse

RR: Relative Risk (RR)

ROB: Risk of Bias

TRST: Triage Risk Screening Tool

*Data extracted from Randomised controlled trials included in Systematic Reviews
