# Supplementary material for: The effectiveness of interventions to reduce adverse outcomes among older adults following Emergency Department discharge: umbrella review
Source: BMC Geriatr. 2022 May 28;22:462. doi: 10.1186/s12877-022-03007-5 (PMC9145107; doi:10.1186/s12877-022-03007-5)
Supplement: Supplementary file 7 — Additional file 7: Supplementary Information 7. Algorithm to Grading of Recommendations, Assessment, Development and Evaluation (GRADE) for all for all outcomes in each systematic review. [file 12877_2022_3007_MOESM7_ESM.docx]

**Supplementary Information 7:**

**Algorithm to Grading of Recommendations, Assessment, Development and Evaluation (GRADE) for all ALGORITHM TO GRADING OF RECOMMENDATIONS, ASSESSMENT, DEVELOPMENT AND EVALUATION (GRADE) for all outcomes in each systematic review.**

**Method of determining number of downgrades to levels of evidence in systematic reviews ^1^**

|  | **Imprecision** | **Risk of bias (trial quality)** | **Inconsistency** | **Risk of bias (review quality)** |
| --- | --- | --- | --- | --- |
| No downgrade (no serious limitations) | ≥200 participants in pooled analysis | ≥75% of participants in pooled analysis judged to have low risk of bias for randomisation and assessor blinding | Heterogeneity, assessed using the I^2^ statistic ≤75% | 4/4 ‘Yes’ responses to AMSTAR 2 questions 2, 4, 5 and 6 (a priori research design, search characteristics, independence of study selection and data extraction) |
| Downgrade 1 level (serious limitations) | 100-199 participants in pooled analysis | <75% of participants in pooled analysis judged to have low risk of bias for randomisation and assessor blinding | Heterogeneity, assessed using the I^2^ statistic >75% | 3/4 ‘Yes’ responses to AMSTAR 2 questions 2, 4, 5 and 6 (a priori research design, search characteristics, independence of study selection and data extraction) |
| Downgrade 2 levels (very serious limitations) | 1-99 participants in pooled analysis |  |  | <3/4 ‘Yes’ responses to AMSTAR 2 questions 2, 4, 5 and 6 (a priori research design, search characteristics, independence of study selection and data extraction). |
| Notes |  | If risk of bias for individual studies was not reported in the review, then it was assumed <75% of participants had low risk of bias | If only one study contributed to the analysis, no downgrade was applied. If I^2^ was not reported, it was assumed to be >75% |  |

Method for applying GRADE level of evidence from number of downgrades determined using the algorithm to GRADE **^1^** :

No downgrade: High level of evidence

Moderate level of evidence: 1 or 2 downgrades

Low level of evidence: 3 or 4 downgrades

Very low level of evidence: 5 or 6 downgrades

**Table 1:** GRADE level of evidence for the effectiveness of Emergency Department (ED) interventions on functional status in older adults discharged from the ED.

| **Citation** | **Relative Effect** | **Imprecision** | **Risk of Bias - Trials** | **Inconsistency** | **Risk of Bias - Review** | **Total Number of Downgrades** | **Level of Evidence** |
| --- | --- | --- | --- | --- | --- | --- | --- |
| Conroy et al. 2011 | MD 0.41, [0.21 to 0.61] favouring intervention via Barthel score (1 RCT). | No downgrade | Downgrade 1 Level- Not reported | No downgrade | 2 downgrades | 3 | Low |
| Fealy et al. 2009 | Two RCTS demonstrated reduced dependence in IADL and short term functional decline. Heterogeneity in the tools used to measure function. | No downgrade | 1 downgrade | 1 downgrade | 2 downgrades | 4 | Low |
| Hastings & Heflin 2005 | 3 RCTS reported a reduction in functional decline at different time points, (range from 4 months to 18 months) and using different measurement tools (dependence with ADLs and IADLs, Barthel). 1 RCT reported no significant difference in functional status . | Downgrade 1 Level - not reported | 1 downgrade | 1 downgrade | 2 downgrades | 5 | Very Low |
| Hughes et al. 2019 | Positive intervention effects were observed in four of the five RCTs Functional status was assessed differently across the studies. | No downgrade | 1 downgrade | 1 downgrade | 1 downgrade | 3 | Low |
| Lowthian et al. 2015 | Measured in 2 RCTS using different tools and at at different time points (6 and 12 months in 1 RCT and 4 months in the other. 1 RCT reporting a greater degree of independence in ADL at 6 months  and ED-CTS group using Barthel tool. At 18 months, deterioration in ADL was reported for both groups. 1 RCT reported no impact on physical (SF 36) with the intervention at either time point. | No downgrade | 1 downgrade | 1 downgrade | 1 downgrade | 3 | Low |

**Table 2:** GRADE level of evidence for the effectiveness of Emergency Department interventions on Quality of Life (QOL)

| **Citation** | **Relative Effect** | **Imprecision** | **Risk of Bias - Trials** | **Inconsistency** | **Risk of Bias - Review** | **Total Number of Downgrades** | **Level of Evidence** |
| --- | --- | --- | --- | --- | --- | --- | --- |
| Conroy et al. 2011 | MD of 0.2 [-1.9 to 2.3] in Physical component of SF 36) and 0.6 [-1.3, to -2.5] in mental component of SF36. | No downgrade | Downgrade 1 Level- Not reported | No downgrade | 2 downgrades | 3 | Low |
| Hastings & Heflin 2005 | 3 RCTs reported no significant change in QOL | Downgrade 1 Level - not reported | No downgrade | 1 downgrade | 2 downgrades | 5 | Very Low |
| Hughes et al. 2019 | There were no statistically significant effects of  the ED interventions on either physical or mental health–  related QOL at any time point in 2 RCTs. | No downgrade | 1 downgrade | 1 downgrade | 1 downgrade | 3 | Low |

**Table 3:** GRADE level of evidence for the effectiveness of Emergency Department interventions on Mortality

| **Citation** | **Relative Effect** | **Imprecision** | **Risk of Bias - Trials** | **Inconsistency** | **Risk of Bias - Review** | **Total Number of Downgrades** | **Level of Evidence** |
| --- | --- | --- | --- | --- | --- | --- | --- |
| Conroy et al. 2011 | RR 0.92, 95% CI [.55 to 1.52], p=0.767. I² = 0%. | No downgrade | Downgrade 1 Level- Not reported | No downgrade | 2 downgrades | 3 | Low |
| Hastings & Heflin 2005 | 3 RCTS showed no effect of ED intervention on mortality | Downgrade 1 Level - not reported | Downgrade 1 Level | Downgrade 1 levels | Downgrade 2 levels | 5 | Very Low |
| Karam et al. 2015 | 2 RCTS reported no effect of ED intervention on mortality | No Downgrade | Downgrade 1 Level -not reported | Downgrade 1 Level | Downgrade 2 Levels | 4 | Low |
| Lowthian et al. 2015 | OR 1.01. 0.70-1.47, p= 0.94  I²= 0% | No Downgrade | Downgrade 1 Level | No downgrade | Downgrade 1 level | 2 | Moderate |

**Table 4:** GRADE level of evidence for the effectiveness of Emergency Department interventions on Patient Experience

| **Citation** | **Findings/Relative Effect** | **Imprecision** | **Risk of Bias - Trials** | **Inconsistency** | **Risk of Bias - Review** | **Total Number of Downgrades** | **Level of Evidence** |
| --- | --- | --- | --- | --- | --- | --- | --- |
| Berning et al. 2020 | There was significant heterogeneity in the tools used to measure patient experience. Improved patient experience was noted from some department wide interventions. | No downgrade | Downgrade 1 Level | Downgrade 1 Level | Downgrade 1 Level | 3 | Low |
| Fealy et al. 2009 | There was no significant effect on the effectiveness of ED intervention to improve patient satisfaction. | No downgrade | Downgrade 1 Level- Not reported | Downgrade 1 Level | Downgrade 2 Levels | 4 | Low |
| Hastings & Heflin 2005 | 3 RCT showed no effect of ED intervention on patient experience | Downgrade 1 Level - not reported | No downgrade | Downgrade 1 Level | Downgrade 2 Levels | 4 | Very Low |
| Hughes et al. 2019 | 4 RCTs reported mixed effects with no statistically significant effects on patient experience in 2 RCTs using a range of continuous outcome measures and unnamed instruments. | No Downgrade | Downgrade 1 Level | Downgrade 1 level | Downgrade 1 level | 3 | Low |

**Table 5: GRADE level of evidence for the effectiveness of Emergency Department interventions on Emergency Department return visits**

| **Citation** | **Relative Effect** | **Imprecision** | **Risk of Bias - Trials** | **Inconsistency** | **Risk of Bias - Review** | **Total Number of Downgrades** | **Level of Evidence** |
| --- | --- | --- | --- | --- | --- | --- | --- |
| Conroy et al., 2011 | RR 0.95 [0.83, 1.08].  I²=42% | No downgrade | Downgrade 1 Level-Not reported | No downgrade | Downgrade 2 levels | 3 | Low |
| Fealy et al., 2009 | Mixed reporting on both decreased ED return visits in some RCT and increased in other RCTS. | No downgrade | Downgrade 1 Level- Not reported | Downgrade 1 Level | Downgrade 2 levels  2 downgrades | 4 | Low |
| Karam et al., 2015 | 3 RCTs reported no effect of ED interventions on ED revisits. | No downgrade | Downgrade 1 Level (not reported) | Downgrade 1 Level | Downgrade 2 Levels | 4 | Low |
| Hastings & Heflin 2005 | One RCT reported an increase in ED readmissions, 1 RCT reported a decrease in ED return visits at one month and 18 months. 2 RCTs reported no change. | Downgrade 1 Level - not reported | No downgrade | Downgrade 1 Level | Downgrade 2 Levels | 4 | Low |
| Hughes et al. 2019 | RR = 1.13; 95% CI = .94 to 1.36 | No downgrade | No downgrade | No downgrade | Downgrade 1 level | 1 | Moderate |
| Lowthian et al 2015 | OR: 1.32, 95% CI: 0.99 to 1.76, I²=0% | No downgrade | Downgrade 1 Level | No Downgrade | Downgrade 1 Level | 2 | Moderate |
| Malik et al. 2018 | OR: 1.03, 95% CI: 0.84, 1.26 I²=62% | No downgrade | Downgrade 1 Level | Downgrade 1 Level | Downgrade 2 Levels | 4 | Low |

**Table 6: GRADE level of evidence for the effectiveness of Emergency Department interventions on Hospital Admissions**

| **Citation** | **Relative Effect** | **Imprecision** | **Risk of Bias - Trials** | **Inconsistency** | **Risk of Bias - Review** | **Total Number of Downgrades** | **Level of Evidence** |
| --- | --- | --- | --- | --- | --- | --- | --- |
| Hastings & Heflin 2005 | There were mixed results in service utilisation with one RCT showing reduction in hospital admission at 30 days and emergency hospital admission at 18 months. 2 RCTS showed no effect on admission rates. | Downgrade 1 Level - not reported | Downgrade 1 Level - not reported | Downgrade 1 Level | Downgrade 2 Levels | 5 | Very Low |
| Hughes et al. 2019 | Relative risk [RR] = .96; 95% CI = .51-1.83*. I*² = 63.25% | No downgrade | Downgrade 1 level | No downgrade | Downgrade 1 level | 2 | Moderate |
| Karam et al. 2015 | 3 RCTS reported mixed effects with 1 RCT showing a reduction in hospital admissions and 1 RCT showing no change with the third RCT reporting a slight change in the intervention group. | No downgrade | Downgrade 1 Level (not reported) | Downgrade 1 Level | Downgrade 2 Levels | 4 | Low |
| Lowthian et al. 2015 | OR 0.89, 95% CI: 0.65 to 1.21. *. I*² =0% | No downgrade | Downgrade 1 Level | No downgrade | Downgrade 1 Level | 2 | Moderate |
| Malik et al. 2018 | OR: 0.84, 95% CI: 0.70 to 1.02,  *I*² =35% | No downgrade | Downgrade 1 Level | Downgrade 1 Level | Downgrade 2 Levels | 4 | Low |

**Table 7: GRADE level of evidence for the effectiveness of multifactorial falls Emergency Department interventions on Rate of Falls**

| **Citation** | **Relative Effect** | **Imprecision** | **Risk of Bias - Trials** | **Inconsistency** | **Risk of Bias - Review** | **Total Number of Downgrades** | **Level of Evidence** |
| --- | --- | --- | --- | --- | --- | --- | --- |
| Morello et al. 2019 | Rate ratio: 0.78, [95% CI: 0.58 to 1.05} I²= 94% | No downgrade | Downgrade 1 Level | Downgrade 1 Level | Downgrade 2 Levels | 4 | Low |

**Table 8: GRADE level of evidence for the effectiveness of multifactorial falls ED interventions on Number of Fallers**

| **Citation** | **Relative Effect** | **Imprecision** | **Risk of Bias - Trials** | **Inconsistency** | **Risk of Bias - Review** | **Total Number of Downgrades** | **Level of Evidence** |
| --- | --- | --- | --- | --- | --- | --- | --- |
| Morello et al. 2019 | Risk ratio: 1.02, [95%CI: 0.88 to 1.18[, I²= 75% | No Downgrade | No Downgrade | No Downgrade | Downgrade 2 Levels | 2 | Moderate |

**Abbreviations:**

ADL: Activities of Daily Living

CI: Confidence Intervals

ED: Emergency Department

IADL: Instrumental Activities of Daily Living

OR: Odds Ratio

RCT: Randomised controlled trial

RR: Rate Ratio

QOL: Quality of Life

**References:**

1. Pollock A, Farmer SE, Brady MC, et al. An algorithm was developed to assign GRADE levels of evidence to comparisons within systematic reviews. *Journal of Clinical Epidemiology* 2016;70:106-10. doi: <https://doi.org/10.1016/j.jclinepi.2015.08.013>
